# Harnessing beneficial bacteria to remediate antibiotic-polluted agricultural soils: integrating source diversity, bioavailability modulators, and ecological impacts

**DOI:** 10.3389/fmicb.2025.1635233

**Published:** 2025-09-19

**Authors:** Oluwaseyi Samuel Olanrewaju, Cornelius Carlos Bezuidenhout

**Affiliations:** Unit for Environmental Sciences and Management, North-West University, Potchefstroom, South Africa

**Keywords:** beneficial bacteria, bioremediation, food security, sustainable agriculture, one health

## Abstract

Antibiotic contamination in agricultural soils, primarily from manure application and wastewater irrigation, has emerged as a critical threat to food security, environmental health, and public safety due to the proliferation and persistence of antibiotic-resistant genes. This review examines the diverse sources and ecological impacts of antibiotics in soil, including their alteration of microbial community structures, promotion of horizontal gene transfer, and subsequent risks to plant and human health. It further evaluates how soil properties, such as pH, organic matter content, and texture, influence the bioavailability of antibiotics and modulate their degradation dynamics. Emphasis is placed on the bioremediation potential of beneficial bacteria, detailing key mechanisms such as enzymatic biodegradation, biosorption, biofilm formation, and the formation of synergistic microbial consortia capable of utilizing antibiotics as nutrient sources. In addition, the manuscript critically discusses the regulatory, technological, and scalability challenges inherent to deploying microbial bioremediation strategies, including integrating gene editing and systems biology approaches under a One Health framework. By synthesizing molecular insights with environmental and policy considerations, this review provides a comprehensive assessment of current bioremediation strategies and outlines future directions to mitigate the ecological and health risks associated with antibiotic pollution in agricultural ecosystems.

## Introduction

The discovery of penicillin marked the beginning of modern medicine (Hutchings et al., 2019), which has led to antibiotics being used as a basic method of treating diseases in people and animals for decades (Xie et al., 2018). The expansion of large-scale animal production systems has raised the demand for veterinary antibiotics, which are used not only to treat infections but also to prevent disease and promote growth. Many veterinary antibiotics are supplied in animal feed at subtherapeutic levels to promote growth (Gonzalez Ronquillo and Angeles Hernandez, 2017; Caneschi et al., 2023). However, many antibiotics are excreted intact or as active metabolites because they are not entirely absorbed (Tiedje et al., 2023). Antibiotic use in farm animals creates an environment that promotes the formation of antibiotic-resistant bacteria (ARBs), which spread through manure and the surrounding ecosystem.

Agricultural soil is crucial to preserving food security and ecological balance. It is a critical resource for crop production, influencing crop yield, quality, and environmental health. The United Nations Sustainable Development Goals (SDGs) emphasize the need for sustainable agricultural practices in ensuring food security, especially considering the world’s growing population and the increasing constraints of climate change. Among the many challenges confronting agricultural systems, antibiotic contamination poses a significant threat to soil health and food safety, raising concerns about the emergence of antibiotic-resistance genes (ARGs) in agricultural ecosystems. The SDGs, particularly goal 2, seek to eliminate hunger, ensure food security, and promote sustainable agriculture by 2030. This goal acknowledges that sustainable agriculture is critical to ensuring food security and promoting nutrition. However, the presence of antibiotics in agricultural soils, commonly caused by manure application and the use of wastewater for irrigation, affects this goal. The widespread use of antibiotics in livestock farming has resulted in the accumulation of these substances in the environment, which can harm soil microbiota and disrupt essential soil functions like nutrient cycling and organic matter decomposition (Udikovic-Kolic et al., 2014; Christou et al., 2017; Checcucci et al., 2020). Beneficial microbes that have developed resistance can spread through the food chain to humans through the consumption of affected crops (Pepper et al., 2018; Lima et al., 2020; Fatoba et al., 2021). Studies have demonstrated manure to be a hotspot for spreading ARGs, promoting horizontal gene transfer among soil bacteria (Deng et al., 2024; Wang S. et al., 2024). Antibiotics in soil alter microbial community structure and functional capabilities, potentially reducing soil fertility and increasing vulnerability to pests and diseases (Yang et al., 2016; Cycon et al., 2019). The persistence of these antibiotics in the soil is affected by environmental factors such as soil type, pH, and organic matter content, thereby affecting their bioavailability and degradation (Li et al., 2022; Liu et al., 2022b; Cui et al., 2023). Antibiotic resistance spreading through the food chain is a major public health concern, as infections caused by resistant bacteria are often more challenging to treat, resulting in increased morbidity and mortality (Han et al., 2022; Elder et al., 2023). Hence, there is a critical need for integrated methods to address antibiotic usage in agriculture, promote sustainable farming techniques, and protect soil health to secure food for future generations.

One of the sustainable solutions to combat this issue is the use of beneficial microbes. Beneficial microbes offer an environmentally friendly and sustainable solution for detoxifying antibiotics in agricultural soil. The effect of the increased concentrations of these antibiotics on soil microbial abundance, diversity, and communities, as well as microbial functions and processes in the soil, can be ameliorated by microbial bioremediation. In relation to antibiotic stress, beneficial microbes have been reported to improve soil health and alleviate plant stress through various mechanisms, such as siderophore production, biotransformation, biosorption, and biodegradation. Many soil contaminants, including antibiotics, pesticides, heavy metals, oil spillage, etc., have been biodegraded using beneficial microbes. For instance, the bacterium *Cupriavidus metallidurans* strain MSR33 has demonstrated significant potential in remediating mercury-contaminated soils, showing the ability to tolerate heavy metals and positively influence the nitrogen cycle in the soil ecosystem (Bravo et al., 2020). In another study by Xu J. et al. (2021), engineered strains were developed to enhance the degradation of p-nitrophenol through specific enzymatic pathways, which are crucial for breaking down complex organic molecules. Therefore, beneficial bacteria can be used to mitigate the effects of these contaminants. In addition to their bioremediation properties, they help improve soil and plant health, promoting plant growth and improving food security (Olanrewaju et al., 2017, 2024a).

Since the increased accumulation of antibiotics on agricultural soil is fast becoming a cause for serious concern because of their detrimental effect on soil microbiome and their role in increasing antibiotic resistance, it is of paramount interest to find a way to alleviate these impacts using an eco-friendly and sustainable approach. Keeping this in mind, this review attempts to present an environmentally sustainable approach to mitigating the effects of these antibiotics on agricultural soil, soil microbial community, and, ultimately, plant health.

## Sources and ecological impacts of antibiotics on agricultural soil

### Sources of antibiotics in soil

Anthropogenic activities are the primary sources of antibiotics in the soil. Human activities have been the primary cause of major disasters such as climate change, flooding, loss of agricultural soil, etc., affecting the environment. Antibiotics are meant to address various medical concerns in humans and animals. However, some antibiotics find their way into agricultural soils in their active forms, which is a serious cause for concern. We look at the sources of antibiotics in agricultural soil. Antibiotics in agricultural settings have complex and significant ecological effects across several environmental compartments, including soil and water (Table 1). Antibiotics used extensively in crop and animal production have generated questions about their environmental persistence and possible disturbance of the ecological equilibrium.

**Table 1:** Sources of antibiotics in agricultural farms.

Source	Antibiotic origin	Pathway into soil	Comments	References
Direct crop application	Agricultural use (e.g., streptomycin, oxytetracycline) on fruit trees	Spray residues landing on soil or uptake by roots.	High impact in treated orchards, minimal globally (few countries, few antibiotics). Regulatory: tightly controlled (EPA registrations) in use countries.	Taylor and Reeder (2020), Verhaegen et al. (2023), and Batuman et al. (2024)
Livestock manure and slurry	Veterinary uses (therapeutic, prophylactic, growth promotion)	Spreading of animal manure or lagoon effluent on fields.	Largest source by volume. Livestock manure often contains heavy residues. Drives soil ARGs. Regulations focus mainly on pathogens/metals, not drugs.	Cycon et al. (2019), Zalewska et al. (2021), Frey et al. (2022), and Matamoros et al. (2022)
Wastewater irrigation	Municipal wastewater	Application of treated or untreated effluent to crops/fields.	Significant in arid regions (Middle East, Africa, South Asia). Antibiotics (e.g., erythromycin, tetracycline) detected in irrigated soils. Few guidelines for pharmaceuticals.	Gatica and Cytryn (2013), Bougnom et al. (2020), Leiva et al. (2021), and Mohy et al. (2023)
Biosolids/sludge application	Sewage treatment plants	Land-spreading of treated sewage sludge or biosolid fertilizers.	Moderate source, antibiotic levels much lower than manure. Provides chronic exposure and metal co-selection. Regulated for pathogens/heavy metals (not specific to antibiotics).	Cycon et al. (2019), Lu et al. (2021), and Qin et al. (2022)
Aquaculture runoff/leaching	Fish farming antibiotics	Discharge or leakage of pond effluent into fields or waterways.	Important near intensive aquaculture. Pond sediments accumulate drugs. Can contaminate irrigation water. Regulatory controls vary, heavy use in Asia.	Kraemer et al. (2019), Dong et al. (2021), Thiang et al. (2021), Shi et al. (2022), and Yi et al. (2025)
Airborne deposition (dust)	Feedlot dust (CAFOs), emissions from pharma plants	Wind transport and fallout of contaminated dust/aerosols onto soil	Emerging concern. Feedlot dust carries antibiotics and ARGs. They can spread contamination meters–kilometers downwind and there are no direct regulation.	McEachran et al. (2015) and Gwenzi et al. (2022)
Pharma manufacturing waste	Antibiotic factories waste	Discharge of untreated or partially treated industrial effluent to land/river.	Potentially highest local concentrations. Documented “superbug” hotspots downriver of plants. Historically unregulated in LMICs, new limits emerging. Can sterilize local soils.	Laxminarayan and Chaudhury (2016) and Kraemer et al. (2019)
Composted wastes	Manure/sludge-derived composts	Application of compost to fields.	If composting is incomplete, residual antibiotics remain. Compost use in agriculture can thus introduce drugs similar to manure. Proper high-temp composting reduces load but does not eliminate all classes.	Zhang M. et al. (2019), Ezugworie et al. (2021), and Qian et al. (2022)

The sources of antibiotics in the agricultural field are multifaceted and involve various practices that contribute to the presence of these compounds in the environment (Table 1). Antibiotics in agriculture primarily stem from their application in crop management through direct application, manure application (Muhammad et al., 2020; Khmaissa et al., 2024), and wastewater irrigation (Li T. et al., 2024; Phan et al., 2024), which raises significant concerns regarding antibiotic resistance and environmental health.

One of the primary sources of antibiotics in agriculture is the use of these compounds in livestock. Antibiotics are administered to animals for therapeutic purposes, growth promotion, and disease prevention. Reports indicate that agricultural antibiotic use accounts for a substantial portion of total antibiotic production, with estimates suggesting that it may represent up to half of all antibiotics produced in the United States (Looft et al., 2012). This extensive use creates a reservoir of ARB and ARGs in animal waste, potentially contaminating soil and water systems (Williams-Nguyen et al., 2016; Zhao et al., 2023). Manure application from livestock operations to fields is a direct pathway for introducing these antibiotics and their associated resistance traits into agricultural ecosystems (Udikovic-Kolic et al., 2014; He et al., 2020).

In addition to livestock, the use of antibiotics in crop production is another significant source. Some antibiotics, such as oxytetracycline and streptomycin, are applied to control bacterial diseases in plants, the uptake of these antibiotics by crops can lead to their accumulation in edible plant tissues, raising concerns about food safety and potential health risks to consumers (Yin et al., 2023).

Furthermore, applying treated wastewater for irrigation, which often contains residual antibiotics, introduces additional sources of these compounds into agricultural soils (Sorinolu et al., 2021) (Table 1). The presence of antibiotics in irrigation water can stem from municipal wastewater treatment plants that discharge effluents containing various pharmaceuticals, including antibiotics, into water bodies used for agricultural purposes (Khan et al., 2020; Gworek et al., 2021).

In addition to direct application, manure application, and wastewater irrigation as major sources of antibiotics in agricultural farms, other not-so-common/reported sources include biosolids, sludge, compost, and pharmaceutical waste application (Table 1).

### Ecological impacts of antibiotics on agricultural soil

**Figure 1:**
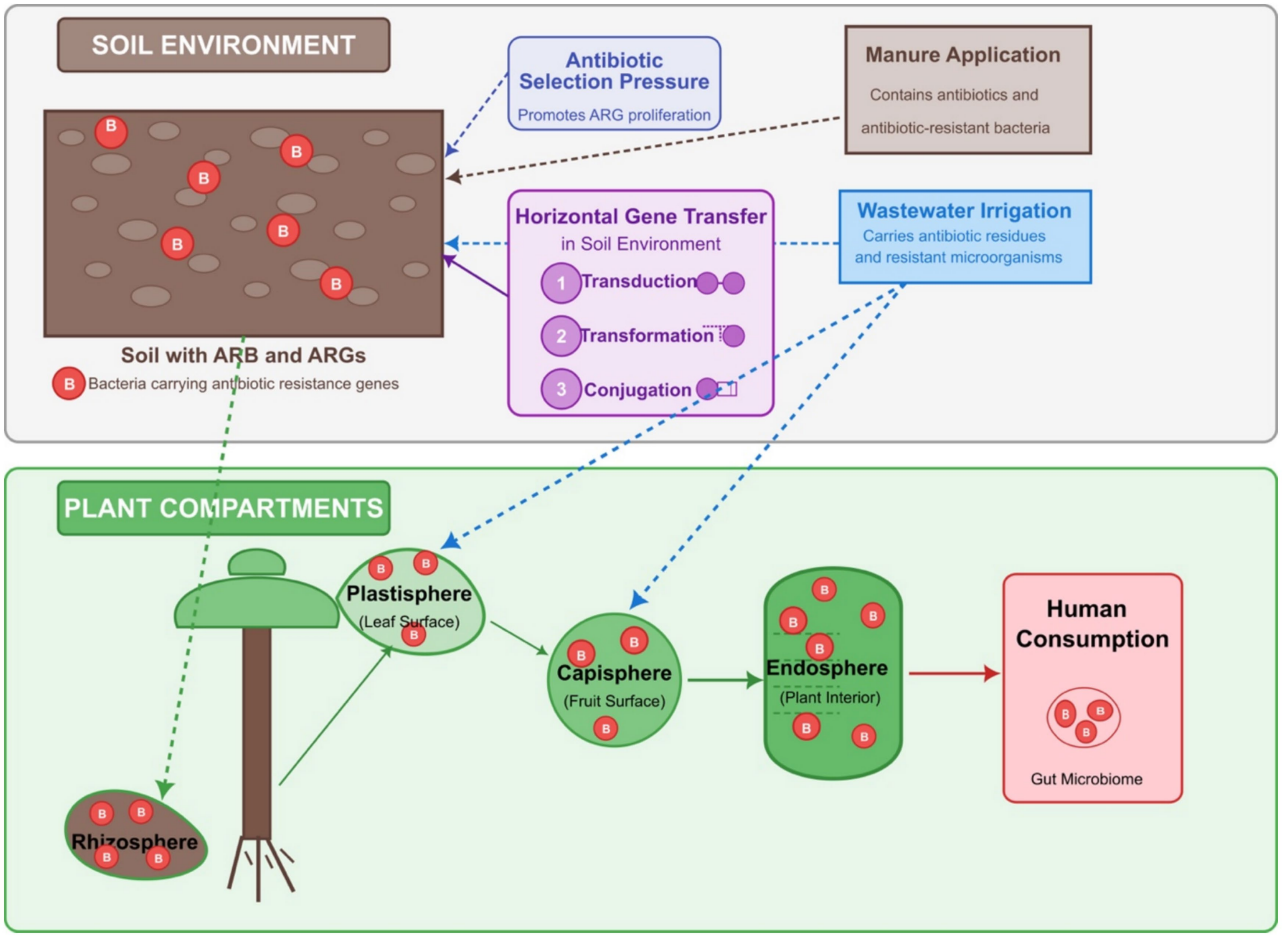
The plant microbiome’s role in antibiotic resistance gene (ARG) transmission through the food chain. The upper section illustrates the soil environment containing ARG-carrying bacteria subjected to selection pressure from antibiotics introduced via manure application and wastewater irrigation. Horizontal gene transfer mechanisms (conjugation, transformation, and transduction) facilitate ARG spread in soil. The lower section details plant compartments, including rhizosphere (root zone), plastisphere (leaf surface), capisphere (fruit surface), and endosphere (plant interior) that harbor ARG-carrying bacteria. Directional arrows demonstrate how bacteria migrate from soil to plant surfaces and eventually into endospheric compartments.

The environmental persistence of antibiotics is a critical factor that exacerbates their impact. Studies have shown that antibiotics can remain in agricultural soils for extended periods, mainly when introduced through manure or sludge (Ghirardini et al., 2020; Buta et al., 2021) (Figure 1). This persistence facilitates the continuous selection of antibiotic-resistant bacteria and contributes to the spread of ARGs within the soil microbiome (Kaviani Rad et al., 2022). The interaction between antibiotics and soil contaminants, such as heavy metals and organic fertilizers, further complicates the dynamics of antibiotic resistance in agricultural settings (Topp et al., 2018). Moreover, the role of agricultural practices in disseminating antibiotic resistance cannot be overstated. The misuse and overconsumption of antibiotics in agriculture have exerted selective pressures on microbial communities, driving the evolution of resistance (Sanderson et al., 2018). The presence of high levels of antibiotic resistance in both urban and rural soils highlights the widespread nature of this issue, indicating that agricultural practices significantly contribute to environmental resistance (Osbiston et al., 2021). The connection between antibiotic use in agriculture and the emergence of resistant strains of bacteria directly threatens public health, as these pathogens can be transmitted to humans through the food chain (Iwu et al., 2020; Samreen et al., 2021) (Figure 1). The implications of antibiotic use in agriculture extend beyond the immediate agricultural environment. ARGs are transmitted from agricultural settings to human populations through various pathways, including consuming contaminated food products, direct animal contact, and environmental exposure. The interconnectedness of agricultural practices and human health underscores the need for comprehensive strategies to mitigate the risks associated with antibiotic resistance. This includes implementing better antibiotic stewardship practices, reducing unnecessary antibiotic use in livestock, and enhancing the monitoring of antibiotic residues in agricultural products (Topp et al., 2018).

The occurrence of antibiotics in soil and water systems has been reported in various studies (Bilal et al., 2020; Kovalakova et al., 2020; Lyu et al., 2020; Snow et al., 2020; Jia et al., 2023), with studies indicating that these compounds can persist and accumulate, posing risks to non-target organisms and disrupting essential ecological processes (Du and Liu, 2012; Danner et al., 2019) (Table 2). For instance, it is reported that the limited understanding of the ecotoxicological relevance of antibiotics, particularly in agricultural organic fertilizers and plant-based products, can lead to unintended exposure to various organisms (Du and Liu, 2012). Introducing antibiotics into the environment can significantly impact microbial communities, which are critical in nutrient cycling and decomposition of organic matter. In their study, Danner et al. (2019), emphasized that non-target organisms, integral to ecological processes, are inevitably exposed to antibiotics when these substances enter surface waters. This exposure can lead to shifts in microbial community structure, resulting in decreased biodiversity and altered ecosystem functions. The disruption of microbial communities can hinder nutrient cycling processes, such as nitrification and denitrification, which are vital for maintaining soil fertility and water quality (Ding and He, 2010; Cycon et al., 2019). In addition, the presence of antibiotics in agricultural settings has been linked to the emergence and proliferation of ARB and ARGs. The use of antibiotics in livestock farming creates selective pressure that fosters the development of resistance among microbial populations. The environmental pollution caused by antibiotic residues is particularly concerning, as it can spread resistant strains into the food chain and water supply, posing a significant public health risk (Kraemer et al., 2019; Wu et al., 2022). The impact of antibiotic pollution extends beyond microbial communities to include aquatic and terrestrial organisms. Studies have shown that exposure to sub-lethal concentrations of antibiotics can induce physiological and behavioral changes in various species. Such findings underscore the need for comprehensive assessments of the ecological risks associated with antibiotic use in agriculture. In addition to direct effects on organisms, antibiotics can interact with other environmental pollutants, leading to synergistic or antagonistic effects that complicate risk assessments. The study by Kraemer et al. (2019) highlights the potential for antibiotic pollution to influence human health through environmental pathways, emphasizing the importance of understanding the interactions between antibiotics and other contaminants. The cumulative effects of multiple antibiotics in the environment can lead to increased toxicity, further exacerbating the ecological impacts.

**Table 2:** Effects of antibiotics on soil microflora.

Antibiotics	Environment	Methods used	Reported effects	References
Cephapirin benzathine and Pirlimycin HCl	Common-garden field plots amended with manure from antibiotic-treated vs. untreated cattle	16S and ITS sequencing, microbial growth/Carbon use efficiency (CUE)	Increased respiration, decreased microbial CUE, and shift toward fungal dominance	Wepking et al. (2019)
Cephapirin benzathine and Pirlimycin HCl	Field plots with repeated manure from antibiotic-treated cattle	16S and ITS sequencing and function assays, ARG profiling	Legacy shifts in microbial community and physiology persisted after cessation.	Shawver et al. (2021)
Cephapirin and Pirlimycin	Lab microcosms with manure from antibiotic-treated cattle under moisture regimes	Respiration, 16S community profiles, moisture × antibiotic interaction tests	Cephapirin increased respiration while pirlimycin decreased respiration, there was also shift in community composition which correlated with moisture.	Shawver et al. (2024)
Cephalosporins	Manure-exposed vs. reference grasslands	Soil respiration and growth, ARG quantification	Increased mass-specific respiration (~2.1×).	Wepking et al. (2017)
Chlortetracycline, Tetracycline, and Oxytetracycline	Lab microcosms and agricultural soils	PLFA, β-glucosidase, urease, phosphomonoesterase activities	Effects varied with soil organic matter. CTC strongest inhibitory effects, antibiotic × soil interactions	Santás-Miguel et al. (2021)
Tetracycline	Pot experiment	16S and ITS sequencing	Concentration-dependent shifts, fungal diversity increased at intermediate dose. Rhizosphere-specific responses were evident.	Zheng et al. (2020)
Oxytetracycline	Soil and hydroponic lettuce systems	Plant traits, qPCR of *tetA* and *tetX*, culture-based isolates	OTC encouraged ARGs. Vertical migration of ARGs to plant tissues detected.	Danilova et al. (2023)
Chlortetracycline, Sulfamethazine, Tylosin, Pirlimycin, and Cephapirin	Small-scale composting of beef and dairy manures	LC–MS quantification	No negative impact on composting efficacy by antibiotics. There was near-complete removal of sulfamethazine and pirlimycin, poor tylosin removal	Ray et al. (2017)
Pirlimycin and Cephapirin	Field plots amended with raw manure or compost during vegetable cultivation	Culturable fecal coliforms, resistance phenotyping	Resistant coliforms recoverable post-application, persistence varied by class of antibiotics.	Wind et al. (2018)
Ciprofloxacin, Enrofloxacin, and Sulfamethoxazole	Raw wastewater-irrigated urban farms	LC–MS for residues, shotgun metagenomics	Increased ARG richness and abundance. Increased shifts in functions and phyla. ARGs associated with higher ciprofloxacin, enrofloxacin, SMX concentrations	Bougnom et al. (2020)
Cephapirin, Pirlimycin, Chlortetracycline, Sulfamethazine, and tylosin	Resistomes in manures/composts, implications for amended soils	Shotgun metagenomics, resistome risk metrics	Composting reduced total ARGs. There was a dominant effect of composting over antibiotic timing	Keenum et al. (2021)

## Effect of antibiotics on ARGs, ARBs, soil microbiome, and plant health

When animal-administered antibiotics find their way into the soil through manure application, they place greater pressure on ARG selection because of their prolonged stay in the soil. Although they are in small doses, the impact of the duration of exposure on ARG and the microbiome is more adverse than their impact on animals because the duration of exposure in animals is short. Studies have been conducted to show the effects of antibiotic exposure to ARGs in soil, for example, a study by Zhu et al. (2013), investigates the prevalence of ARGs in soils associated with large-scale Chinese swine farms. Using a high-throughput quantitative PCR, the study reported 149 unique ARGs, many significantly enriched compared to control soils, with fold increases reaching as high as 28,000. Similarly, studies have shown that using antibiotics in livestock creates selective pressures that favor the proliferation of resistant bacteria, leading to increased ARG abundance in fecal matter (Storteboom et al., 2010). The continuous application of antibiotics in agriculture selects resistant strains and facilitates horizontal gene transfer among microbial communities, amplifying the spread of resistance traits (Gromala et al., 2021; Larsson and Flach, 2022).

Manure is often nutrient-rich and serves as a reservoir for various antibiotics, which can significantly influence the soil microbiome. Studies have shown that introducing manure into soil can increase the abundance of ARGs, as these genes can be transferred among microbial populations through horizontal gene transfer mechanisms. For instance, the study by Zhu et al. (2019) demonstrated that the trophic transfer of ARGs occurs within soil detritus food chains, indicating that organisms feeding on manure can acquire these resistance genes, amplifying their presence in the soil microbiome. Furthermore, Negreanu et al. (2012) noted that while the application of manure with high levels of ARBs initially increases resistance in the soil microbiome, this resistance often returns to baseline levels within six months, suggesting a dynamic equilibrium influenced by environmental factors and microbial interactions. The persistence and spread of ARGs in soil are influenced by various factors, including the type of manure applied and the microbial community structure. Hilaire et al. (2022) found that subsurface manure injection can reduce the surface transport of ARGs but may create localized hotspots of resistance within the soil. This phenomenon underscores the complexity of manure application practices and their varying impacts on soil health. Additionally, Xu H. et al. (2021) highlighted the role of soil bacteria as carriers for plasmids that harbor ARGs, facilitating their transmission to plant endophytic bacteria, which can further propagate resistance within plant microbiomes. This interaction between soil and plant microbiomes is crucial, as it can lead to the establishment of resistant strains in crops, posing potential risks to food safety and human health.

The soil microbiome itself is significantly affected by manure application, which can alter microbial diversity and community composition. Banerjee and Heijden emphasized that urban soil microbiomes often contain higher levels of ARGs and genes associated with human pathogens, likely due to anthropogenic influences such as manure application (Banerjee and van der Heijden, 2023). The introduction of manure can enhance microbial diversity, as reported in the study by Sun et al. (2021), which reported that manure application introduced specific resistance genes to surface soils, thereby reshaping the microbial landscape. This alteration in microbial communities can have cascading effects on soil health, nutrient cycling, and plant interactions, ultimately influencing agricultural productivity. The impact of manure-derived ARGs on plant health is a critical area of concern. Research indicates that ARBs in the soil can affect plant growth and health, potentially leading to reduced crop yields. Muurinen et al. (2017) reported that while manure application does not permanently alter the resistance profile of soils, the cyclic changes in the resistome can affect plant health over time. Similarly, Jauregi et al. (2021) also reported that the mobility of antibiotic resistance from manure to soil and vegetable microbiomes is a significant risk factor, as it can lead to the contamination of food crops with resistant pathogens. This highlights the need for careful management of manure application to mitigate risks associated with antibiotic resistance in agricultural systems. Environmental factors and management practices further complicate the dynamics of ARGs and ARBs in manure-amended soils. For example, Wang E. et al. (2024) noted that long-term manure application can change bacterial communities in the rhizosphere, which may influence plants’ uptake of contaminants such as heavy metals and pathogens. This interaction between soil microbiomes and plant health is critical, as it can determine the overall resilience of agricultural systems to stressors such as disease and nutrient deficiencies. Additionally, heavy metals in manure can exacerbate the risks associated with antibiotic resistance, as these metals can co-select for resistant strains.

Besides manure application, antibiotics can be introduced into agricultural soil through irrigation. About 70% of freshwater globally is being used for irrigation, meaning that water demand will increase in the near future (Sharma et al., 2020). This has made wastewater irrigation a valuable resource for water shortage, especially in developing countries, and this may be more common in the near future (Tian et al., 2021). Although wastewater irrigation offers positive advantages as it contains nutrients important for soil fertility and plant health, it also contains pollutants (Kodesova et al., 2024), including heavy metals (Ahmed et al., 2023; Ugulu et al., 2024) and antibiotics (Mehanni et al., 2023; Mohy et al., 2023), which are environmental risks. Antibiotics in wastewater can increase the dominance of ARBs through selective pressures and horizontal gene transfers (Guo et al., 2017). Therefore, this poses a health risk to humans as these strains can be acquired through the food chain. As a matter of concern, reported exposure to ARBs through the food chain from agricultural soils irrigated (Rahman et al., 2021; Geng et al., 2022).

## Potential risks of antibiotics in agricultural soil to food security and human health

The soil consists of diverse microbial communities, and bacteria have been reported to be the most abundant (Olanrewaju et al., 2017). Bacteria carrying ARGs are also present in the soil. However, the introduction of antibiotics through manure application and wastewater irrigation systems, albeit in small concentrations, for a prolonged period, exerts selective pressure on the soil microbiome (Banerjee and van der Heijden, 2023). In response, as a survival mechanism, the soil microbial community develops resistance to these antibiotics to protect itself. This increases the presence of ARBs and ARGs in the soil microbiome. In addition, the manure and wastewater can carry ARBs and ARGs, which are directly transferred to agricultural soil (Mohy et al., 2023; Zhang Y. et al., 2023). Hence, although beneficial to plant growth, these activities are key to disseminating ARBs and ARGs in agricultural soil. However, studies of the potential spread of antibiotic resistance in the environment have mostly focused on the evolution of antibiotic resistomes in soil and wastewater, with little attention paid to the subsequent spread of antibiotic resistance via plant microbiomes. Many studies have investigated the potential dissemination of antibiotic resistance in the environment (including agricultural soil and wastewater) using qPCR, genomics, metatranscriptomics, and metagenomics (D'angelo, 2023; Ferreira et al., 2023; Kim et al., 2023; de Farias et al., 2024; Molale-Tom et al., 2024; Olanrewaju et al., 2024b,c). This indicates the urgent need for proper waste disposal and recycling for sustainable agriculture (Chen et al., 2019).

Soil microbiomes enter the plant endosphere through the root surface. The plant consists of the endosphere and the ectosphere. The endosphere is inside the plant tissues, while the ectosphere is further categorized into the plastisphere (leaf surface), the capisphere (fruit surface), and the rhizosphere (root surface). The most affected plant compartments in the agricultural fields are the rhizosphere, plastisphere, and capisphere, depending on irrigation and manure application. The microbiome in soil finds its way to the rhizosphere, and when the microbiome is abundant in ARBs, these also make their way to the rhizosphere. Likewise, ARBs in irrigation wastewater are attached to the plastisphere and capisphere, the plant’s above-ground parts. Irrigation affects above-ground and below-ground plant compartments, while manure application affects the below-ground part because it is applied directly to the soil. The entry of the microbial community from the ectosphere into the endosphere provides a transfer route for ARBs and ARGs from manure and wastewater to the soil and the plants (Scaccia et al., 2021). This has been reported in many studies, for example, according to the study by Xu H. et al. (2021), soil bacteria can transfer plasmids harboring ARGs to plant endophytic bacteria, especially those belonging to the phylum Proteobacteria, which contains a variety of plant and human pathogens. This transfer risks human health and agricultural output by making these pathogens more antibiotic-resistant and virulent. These transfers have significant ramifications, indicating that soil management techniques may unintentionally contribute to the spread of antibiotic resistance in agricultural systems. In addition, the composition of the soil microbiome plays a crucial role in determining the health of plants (Banerjee and van der Heijden, 2023). The initial soil microbiome was reported on plant health (Wei et al., 2019), with shifts in microbial communities occurring due to root exudates that alter the microbial landscape. Therefore, the soil microbiome’s initial state can influence the types of bacteria that colonize the plant endosphere, including those that may carry ARGs.

Upon entering plants, these ARBs and ARGs can further find their way into humans through consumption. Although the direct transfer of ARBs and ARGs from plants to humans has not been fully substantiated, this possibility is very high, especially in leafy vegetable crops such as spinach, lettuce, etc., which are often consumed uncooked or partially cooked. Even when washed, the endosphere remains unaffected. Hence, antibiotic-resistant endosphere bacteria in the vegetables can be problematic when ingested by humans. Apart from causing issues, they can also transfer ARGs to the human gut microbiome through horizontal gene transfer (HGT) via plasmids and mobile genetic elements (MGE) (Rossi et al., 2014; Mafiz et al., 2021). This is a big cause for alarm from one health perspective because studies have shown that consumption of these vegetables is a possible route of ARGs from the soil microbiome to humans (Zhang Y. J. et al., 2019; Mafiz et al., 2021; Rahman et al., 2021). In addition, apart from taking up ARBs, plants can also take up antibiotics directly from the soil amended with manure or irrigated with wastewater (Azanu et al., 2016; Gu et al., 2021; Tadic et al., 2021), which may also exert selection pressure on the plant endosphere microbiome and increase the possibility of the development of a more resistant microbiome, which can be transferred to humans upon consumption. Due to its findings in many studies, the class I integron gene (*intI1*) and genes encoding transposases are common in harvested vegetables (Freitag et al., 2018; Urra et al., 2019; Zhang Y. J. et al., 2019; Yuan et al., 2022), indicating the possibility of HGT in the phyllosphere. The phyllosphere and rhizosphere may be key areas for HGT in plant and soil habitats because of the high possibility that cells cluster, forming biofilms in the phyllosphere, and bacterial metabolic rates and the mobility of bacteria and MGEs are high in rhizospheres (Chen et al., 2019). Using a functional metagenomic screen of soil-inhabiting bacteria, a significant nucleotide identity (>99%) was observed between resistance cassettes in multidrug-resistant bacteria from soil and those in human pathogens from clinical environments, indicating the occurrence of HGT between these microorganisms (Forsberg et al., 2012). In the screen, two class 1 integrase genes (*intI1*) from the soil bacteria and clinical pathogens were adjacent to the ARGs, facilitating a shared mechanism of HGT between these two bacterial groups. Class 1 integrons, containing the gene *intI1*, play an essential role in integrating multiple ARGs on the same genetic locus, generating multidrug resistance in bacterial genomes. The integrons have been observed as prevalent carriers of multiple ARGs in natural and anthropogenically influenced environments (Guo et al., 2017). Based on a study with archived soils, manure applications substantially increased the abundance of soil *intI1* (Urra et al., 2019).

The interplay between soil microbiomes, agricultural practices, and human health reveals a critical need to rethink soil and plant management approaches. Harnessing beneficial microbes presents a viable, environmentally sustainable solution to counteract the spread of antibiotic resistance while improving plant productivity. The subsequent discussion focuses on the promising role of these microbes in fostering agricultural sustainability.

## One-health synthesis: linking residues, resistance, and mitigation

Antibiotic residues from manure and wastewater often persist in soils at sub-inhibitory levels that both select for resistant bacteria and elevate horizontal gene transfer, including conjugative plasmid transfer across environmental microbiomes. Laboratory work shows that sub-MIC exposures to aminoglycosides, carbapenems, fluoroquinolones, and cephalosporins increase conjugation frequencies, mechanistically supporting resistance exchange at environmental concentrations below MICs. Related studies report fluoroquinolone-driven, dose-dependent increases in RP4 plasmid transfer from *E. coli* to *P. aeruginosa* at sub-MICs, highlighting a realistic pathway for ARG dissemination under low-level exposures (Ding et al., 2022). Beyond antibiotics, heavy metals common in agri-food systems (Cu, Zn, Hg, Cd) act as co-selectors that stabilize and enrich ARG-bearing elements even when antibiotic concentrations are low, reinforcing persistence across soil-crop-animal-human interfaces (Shun-Mei et al., 2018). Field evidence from raw-wastewater-irrigated agriculture shows soils with elevated transferable ARGs, including ESBLs, and community functional shifts, documenting an environmental conduit consistent with the mechanistic data (Bougnom et al., 2020).

Actionable implications follow directly. First, benchmark effluents and manures against predicted no-effect concentrations (PNEC) for resistance selection thresholds, which are typically lower than ecotoxicity PNECs; compound-specific PNEC resistance values have been proposed to guide emission limits (Bengtsson-Palme and Larsson, 2016). Where antibiotic-specific data are lacking, a default target of 0.05 μg L^−1^ has been recommended for antibiotics to minimize selection pressure (Vestel et al., 2022). Second, treated manures in multi-omics field trials show composting reduces total ARGs and resistome risk relative to raw manure, although marker-specific exceptions warrant reporting treatment stage and verifying with sentinel ARGs (Keenum et al., 2021). Together, source control (including co-selectors), evidence-based waste handling, and targeted surveillance at high-risk nodes operationalize a one health response that links mechanistic insight to practical mitigation in agricultural landscapes (Bougnom et al., 2020).

## Bioavailability of antibiotics and bioremediation efficacy: exploring the link

**Figure 2:**
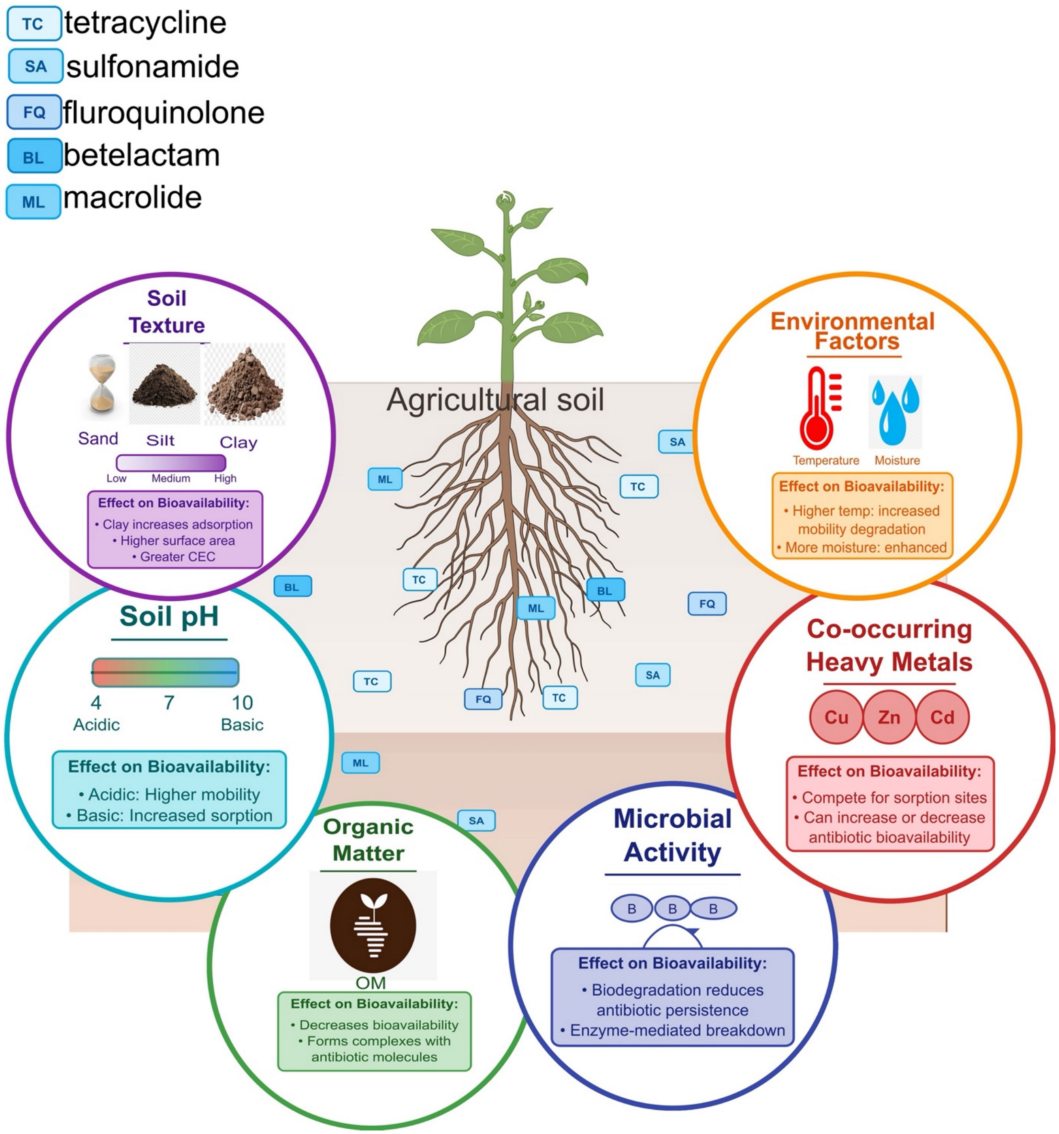
The central soil matrix contains representative antibiotics from different classes (tetracyclines, fluoroquinolones, sulfonamides, macrolides, and beta-lactams). Surrounding this matrix are six key influencing factors: (1) Soil pH—modulating antibiotic mobility through ionization state changes, with acidic conditions increasing mobility and basic conditions promoting sorption for ionizable antibiotics, (2) Organic matter—decreasing bioavailability through complex formation with antibiotics, (3) Soil texture—demonstrating how increasing clay content enhances antibiotic sorption through greater surface area and cation exchange capacity, (4) Environmental factors—showing how higher temperature and moisture can increase both mobility and degradation rates, (5) Co-occurring heavy metals—competing for sorption sites and potentially either increasing or decreasing antibiotic bioavailability, and (6) Microbial activity—contributing to biodegradation processes that reduce antibiotic persistence.

The behavior and fate of antibiotics in the environment are not only determined by their physicochemical properties, which include volatility, lipophilicity, water solubility, and sorption capacity, but also determined by various environmental factors, including the different soil properties such as pH, ionic strength, organic matter content, and cation exchange capacity, and the ecological climatic conditions (Figure 2). As a result of these factors, antibiotics can remain in the environment for a short or extended period. Understanding these interactions is essential in assessing the environmental impact of antibiotic residues, particularly in agricultural settings.

### Effect of soil parameters on antibiotic bioavailability in soil

Soil pH plays a pivotal role in determining the adsorption and desorption of antibiotics. Studies have shown that the adsorption capacity of antibiotics such as ciprofloxacin and trimethoprim varies with soil pH, indicating that lower pH levels can enhance the mobility of these compounds in the soil environment (Rodríguez-López et al., 2024). The ionic state of antibiotics, influenced by pH, affects their solubility and interaction with soil particles. For instance, acidic conditions can increase the solubility of certain antibiotics, thereby enhancing their bioavailability (Pauletto and De Liguoro, 2024). Conversely, higher pH levels can increase adsorption onto soil particles (Yuan et al., 2024), reducing their bioavailability and potential ecological impact.

Organic matter content is another critical factor affecting antibiotic bioavailability. The presence of organic matter causes the formation of complexes between organic molecules and antibiotic compounds. This interaction leads to a decrease in the mobility of antibiotics, thus reducing their bioavailability to soil microorganisms and plants (Li H. et al., 2024). For example, biochar, a form of carbon-rich organic matter, has been shown to significantly reduce the bioavailability of antibiotics in soil by adsorbing these compounds and preventing their uptake by plants (Pan et al., 2023). The effectiveness of biochar in mitigating antibiotic pollution is attributed to its large surface area and the presence of functional groups that facilitate adsorption (Haider et al., 2024; Jia et al., 2024).

Soil texture, which refers to the size distribution of soil particles, also influences antibiotic bioavailability. Soils with a high clay content typically exhibit greater antibiotic adsorption capacities than sandy soils. This is due to the clay particles’ larger surface area and higher cation exchange capacity, which can bind antibiotics more effectively (Jorge et al., 2024). For instance, studies have demonstrated that clay minerals can significantly influence the retention of antibiotics like tetracycline in soil, affecting their bioavailability and persistence (Li S. Y. et al., 2023). The interaction between antibiotics and soil minerals is complex and can be influenced by factors such as ionic strength and competing ions in the soil solution (Xiao et al., 2023).

Other contaminants, such as heavy metals, can also impact the bioavailability of antibiotics in soil. Heavy metals can compete with antibiotics for adsorption sites on soil particles, potentially altering the adsorption dynamics of antibiotics (Nkoh et al., 2024). Additionally, the co-occurrence of heavy metals and antibiotics can lead to synergistic effects that enhance the persistence of both contaminants in the soil environment (Zha et al., 2023). For example, the adsorption of antibiotics can be influenced by heavy metals, which may either enhance or inhibit the mobility of antibiotics depending on the specific interactions involved (Shu et al., 2025).

Microbial communities in the soil are also affected by the bioavailability of antibiotics. Continuous exposure to antibiotic residues can lead to shifts in microbial community composition, which may affect the degradation and transformation of these compounds (Qiu et al., 2023). The physicochemical properties of antibiotics, such as their hydrophobicity and molecular structure, play a crucial role in determining their bioavailability and the ability of soil microorganisms to degrade them (Nkoh et al., 2024). For instance, antibiotics with higher hydrophobicity tend to adsorb more strongly to soil particles, thereby reducing their bioavailability for microbial degradation (Deng et al., 2024).

Moreover, the dynamics of antibiotic bioavailability in soil are influenced by environmental factors such as moisture content and temperature. Increased soil moisture can enhance the mobility of antibiotics by facilitating their transport through soil pores, while higher temperatures may accelerate the degradation processes (Yang et al., 2024). These environmental conditions can significantly alter the bioavailability of antibiotics, impacting their ecological risks and potential for contaminating groundwater and surface water (Luo et al., 2024).

### Linking bioavailability to bioremediation

Bioavailability refers to the extent to which an antibiotic present in soil can be utilized or broken down during bioremediation. When antibiotics are readily accessible, bacteria can degrade them efficiently, thereby contributing to the remediation of soil environments. However, the process may be impeded if they are difficult to access, such as when adhered to soil particles. The increased availability of antibiotics facilitates a more rapid degradation by bacteria, thereby enhancing the efficacy of bioremediation processes. For instance, water-soluble antibiotics present a more accessible option for bacterial utilization and degradation. However, if these antibiotics are firmly adhered to the soil matrix, the accessibility for bacteria diminishes, thereby impeding the remediation process. Research indicates that soil can diminish antibiotic efficacy, resulting in a reduced availability for bacterial interaction.

Bioavailability denotes the degree to which an antibiotic present in soil can be absorbed or engaged with living organisms, especially bacteria. This factor is essential for the process of bioremediation. Bioremediation refers to how microorganisms, particularly bacteria, facilitate the degradation or transformation of environmental pollutants, including antibiotics, into less harmful substances. Antibiotics manifest in multiple forms in the soil environment, whether dissolved in the soil water, adsorbed onto soil particles, or complexed with organic matter. Each of these states significantly influences their bioavailability for bacterial degradation processes.

Antibiotics’ bioavailability is a crucial factor influencing bioremediation efficiency, as bacteria need access to the antibiotics to commence the degradation process. When an antibiotic exhibits high bioavailability, indicating its ready accessibility within the soil solution, bacteria can degrade it effectively, thereby improving the cleanup rates. On the other hand, if it is firmly adsorbed or bound, its bioavailability diminishes, which may impede or restrict degradation processes as fewer bacteria can engage with it.

In addition, antibiotics’ bioavailability directly impacts the rate and efficiency of bioremediation processes. The enhanced bioavailability promotes a more rapid degradation process, allowing bacteria to absorb and metabolize the antibiotic efficiently. For example, antibiotics soluble in water, such as sulfonamides, exhibit greater bioavailability and are consequently more readily degraded by bacterial action. In contrast, less soluble antibiotics like tetracyclines tend to adhere to soil particles, affecting their degradation dynamics, hence, the degradation rate of antibiotics in soil is contingent upon their bioavailability (Cycon et al., 2019). Specifically, adsorbed antibiotics exhibit reduced accessibility for microbial degradation, which may subsequently diminish the efficiency of bioremediation efforts (Hong et al., 2020).

Furthermore, low bioavailability, frequently attributed to robust adsorption to soil constituents, can significantly impede bioremediation efforts by restricting bacterial accessibility. This observation holds significant importance for antibiotics characterized by elevated partitioning coefficients, exemplified by tetracyclines, which tend to associate with soil preferentially. This affinity contributes to their extended persistence and hinders degradation, as discussed by Cycon et al. (2019). Nevertheless, certain bacterial species can surmount low bioavailability by synthesizing biosurfactants or enzymes that promote desorption, thereby augmenting degradation rates. This indicates that the composition and activity of microbial communities play a pivotal role in influencing the effects of bioavailability on bioremediation processes. An intriguing aspect is that low bioavailability may play a role in disseminating ARGs (Li Q. et al., 2024). For example, residual antibiotics in soil, attributed to their low bioavailability, increase the level of soil ARGs by favoring the proliferation of resistant bacterial populations (Chen et al., 2017; Liang et al., 2017), thereby presenting significant ecological risks. The interplay of hindering degradation and the potential dissemination of resistance introduces significant complexities to bioremediation strategies, necessitating meticulous management practices.

### Bioremediation efficacy of beneficial bacteria

Antibiotics have been used extensively in agricultural techniques, which has led to the accumulation of these compounds in soil, resulting in significant environmental problems. The existence of these antibiotics is a cause for great concern since it has the potential to result in the development and dissemination of bacteria that are resistant to antibiotics, which poses substantial dangers to the health of both humans and animals (Guo, 2021). At the same time, there has been a growing interest in employing beneficial bacteria for bioremediation to degrade these compounds. This is a practical approach that has attracted a lot of attention. Their roles and limitations for bioremediation must be better understood for effective utilization.

Bacterial bioremediation represents a highly effective, cost-efficient, and environmentally sustainable approach for removing antibiotic contamination from soil environments, utilizing the metabolic capabilities of specific microorganisms to degrade persistent pharmaceutical compounds (Mokrani et al., 2024). The three distinct mechanisms used by beneficial bacteria in bioremediation processes are bioaugmentation, which involves introducing specialized microbial strains or consortia with enhanced biodegradative capacities, biostimulation, which enhances indigenous microbial activity through nutrient supplementation, and bioattenuation, which relies on natural biological transformation processes. Research has demonstrated remarkable success with specific bacterial strains, such as *Burkholderia cepacia* immobilized on sugarcane bagasse, which effectively degrade tetracycline antibiotics under optimal conditions, including temperatures of 28–43 °C, slightly acidic pH levels (4.5–6.5), and inoculation doses of 15% (Hong et al., 2020). The significance of this approach extends beyond simple contaminant removal, as tetracycline antibiotics, for example, exhibit the highest soil partitioning coefficients among pharmaceutical compounds (Chen et al., 2024), leading to prolonged environmental persistence and serious ecological threats, including alteration of microbial community structures, enhancement of ARG abundance, and bioaccumulation in soil organisms. Furthermore, bacterial bioremediation offers substantial advantages over traditional remediation methods such as adsorption and photocatalytic degradation, which often prove inadequate due to high costs, generation of toxic byproducts, or ecological hazards, making bacterial approaches particularly valuable for addressing the growing environmental challenge of antibiotic contamination that poses long-term threats to ecosystem security and public health.

#### Mechanisms of bioremediation by beneficial bacteria

**Figure 3:**
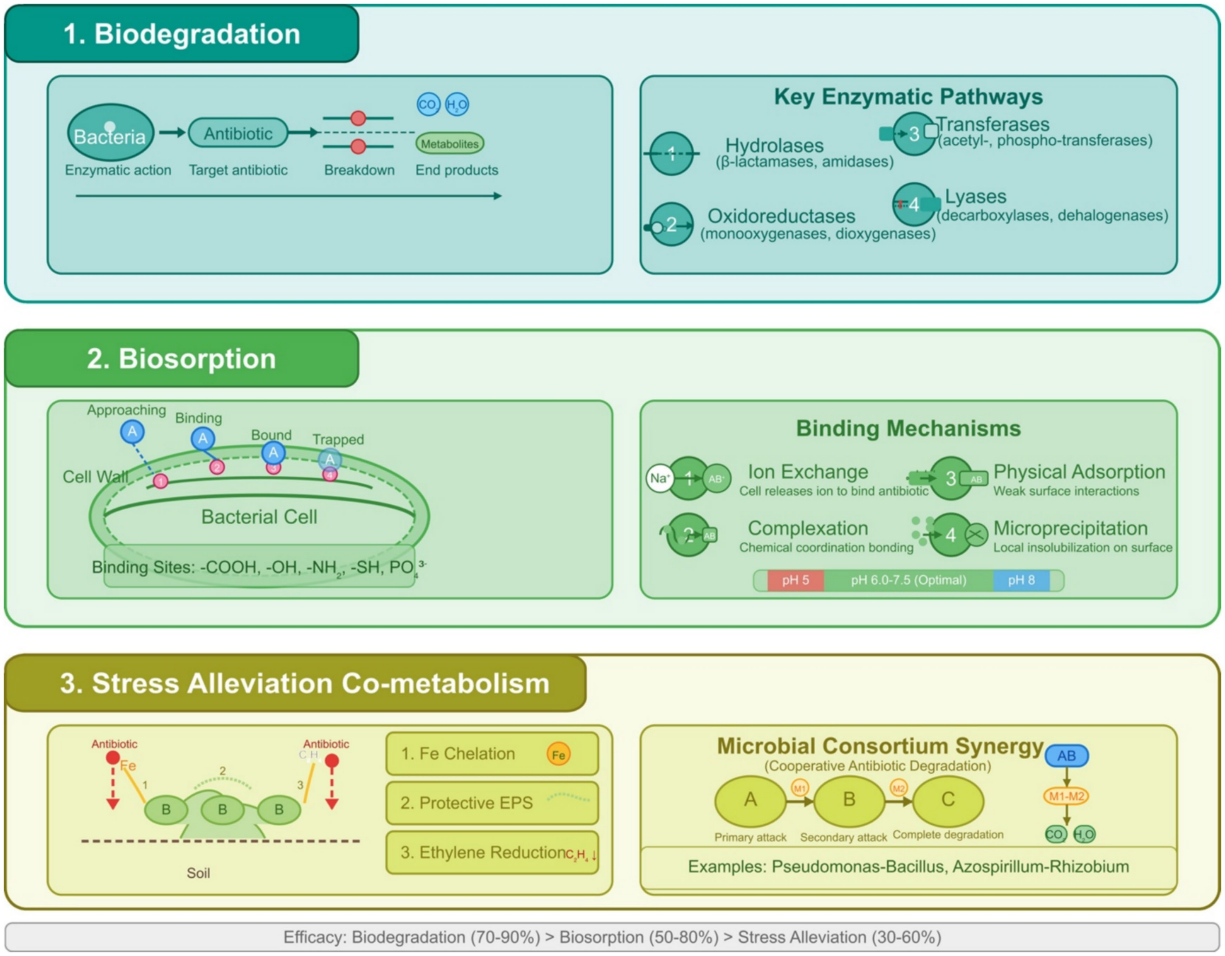
(1) Biodegradation—depicting the enzymatic breakdown of antibiotic molecules into metabolites and ultimately into CO₂ and H₂O through key enzymatic pathways including hydrolases (*β*-lactamases, amidases), oxidoreductases (monooxygenases, dioxygenases), transferases (acetyl-, phospho-transferases), and lyases (decarboxylases, dehalogenases). (2) Biosorption—showing antibiotic adsorption onto bacterial cell surfaces through multiple binding mechanisms, including ion exchange, complexation, physical adsorption, and microprecipitation, facilitated by functional groups (-COOH, -OH, -NH₂, -SH, PO₄^3−^) on bacterial cell walls. (3) Stress alleviation & co-metabolism—illustrating how bacterial products like siderophores, exopolysaccharides, and ACC deaminase mitigate plant stress from antibiotic exposure. At the same time, microbial consortia demonstrate metabolic network synergies that enhance overall degradation capacity.

Manure and wastewater-related antibiotic pollution of agricultural soils helps ARGs to spread and change microbial ecosystems. By producing degrading enzymes, using antibiotics as a nutrient source, producing biofilms, acquiring biodegrading genes through HGT, and participating in cooperative activities (Figure 3; Table 3), beneficial bacteria help reduce this threat.

**Table 3:** Some studies on bacterial bioremediation of antibiotics.

Antibiotics	Bacterial strain	Isolation source	Reported performance	Mechanism/key enzymes	Mode of action	References
Sulfamethoxazole (SMX)	*Microbacterium* sp. BR1	Membrane bioreactor/lab culture	~60% mineralization of 0.1–25 μg/L SMX to CO₂ within 24 h; rapid removal within 2 h	SadA/SadB FMNH₂-monooxygenases; SadC FMN reductase	Biodegradation	Ricken et al. (2017) and Lopez Gordillo et al. (2024)
	*Pseudomonas silesiensis* F6a	Activated sludge (WWTP)	12 products across 4 pathways under aerobic conditions	Oxidative and conjugation steps mapped by LC–MS	Biotransformation	Liu et al. (2022a)
Sulfamethazine (SMZ)	*Microbacterium* sp. C448	SMZ-exposed agricultural soil	Soil microcosms: ≤5.5% mineralization without manure vs. 5.6–19.5% with manure in about 1 month	sadA-encoded antibiotrophy, inducible catabolism	Biodegradation (antibiotrophy)	Billet et al. (2021) and Paris et al. (2023)
Sulfamethoxazole (SMX)	*Ochrobactrum* sp. SMX-PM1-SA1	Environmental isolates	45.2% of 5 mg/L in 288 h	ND	Biodegradation	Mulla et al. (2018)
	*Labrys* sp. SMX-W1-SC11		62.2% of 5 mg/L in 288 h			
	*Gordonia* sp. SMX-W2-SCD14		51.4% of 5 mg/L in 288 h			
Tetracycline (TC)	*Stenotrophomonas maltophilia* DT1	River sediment	89% of 50 mg/L at 30 °C, pH 9	Deamination, denitromethylation, decarbonylation	Biotransformation	Leng et al. (2016)
	*Klebsiella* sp. SQY5	Municipal sludge	Up to 89.66% removal at 80 mg/L	Deamination		Shao et al. (2018) and Shao et al. (2019)
Oxytetracycline (OTC)	*Lysinibacillus* sp. strain 3 + I	Poultry manure	About 85% OTC removal in 7 days	ND	Biodegradation	Sudha et al. (2022)
Ciprofloxacin (CIP)	*Paraclostridium* sp. strain S2	Sulfate-reducing sludge/enrichment	Specific biotransformation rate 1,975.7 ± 109.1 μg g^−1^ CDW h^−1^ at 20 mg/L	Cytochrome P450, dehydrogenases; EPS/adhesion implicated	Biosorption and biotransformation	Fang et al. (2021), Xu et al. (2024), and Zhou et al. (2024)
Erythromycin A (ERY)	*Paracoccus versutus* W7	Sewage sludge	58.5% of 50 mg/L in 72 h at 35 °C, pH 7; complete removal from fermentation residue (100–300 mg/L) in 36–60 h	Erythromycin esterase EreA	Biodegradation	Ren et al. (2023)
Tylosin (TYL)	*Klebsiella oxytoca* TYL-T1	WWTP wastewater	Efficient TYL degradation with lactone ring cleavage; quantitative removal reported (time-course)	Oxidoreductases; ester bond hydrolysis		Zhang T. et al. (2022)
Chloramphenicol (CAP)	*Klebsiella* sp. YB1	Activated sludge (Cd co-stress)	22.4% of 10 mg/L in 48 h	Proposed dechlorination steps under metal stress		Tan et al. (2023)

##### Enzymatic degradation

Enzymatic degradation is a critical process in the bioremediation of antibiotics, where bacteria produce specific enzymes to break down these compounds, making them inert. Bacterial enzymes can break down antibiotic molecules, rendering them inert (Figure 3). The unique genetic composition of the bacterial species involved impacts the efficacy of these enzymes, thereby exposing a complicated relationship between microbial genetics and bioremediation capacity (Elarabi et al., 2023). This method is essential for reducing antibiotic residues in the environment and preventing the spread of antibiotic resistance. For *β*-lactam antibiotics, such as penicillins, β-lactamases hydrolyze the β-lactam ring, inactivating the antibiotic, with over 890 known variants, including extended-spectrum β-lactamases and carbapenemases (Bush, 2010). Tetracyclines are degraded by enzymes like those encoded by the *tetX* gene, which are flavoenzymes that inactivate them through oxidation, as shown with environmental isolates in the study by Gasparrini et al. (2020). Aminoglycosides, like kanamycin, are modified by the enzymes acetyltransferases, adenyltransferases, and phosphotransferases, altering their structure to reduce efficacy, with a specific example being a periplasmic dehydrogenase complex that initiates kanamycin deglycosylation (Chen et al., 2023). Another example is *Arthrobacter nicotianae*, which breaks down tetracyclines (Zhang et al., 2021). General enzymes like oxidoreductases, laccases, and hydrolases are also explored for their role in degrading a broad range of antibiotics, though their specific contributions are less defined (Karigar and Rao, 2011; Bhandari et al., 2021). Understanding these enzymatic mechanisms is crucial for developing effective bioremediation strategies, allowing the selection of appropriate microorganisms or enzymes based on the type of antibiotic and environmental conditions, enhancing cleanup efforts, and reducing environmental impact.

##### Using antibiotics as a nutrient source

Bacteria can harness antibiotics and various pollutants as nutrient sources, specifically for carbon and energy, which facilitates the degradation of these compounds and mitigates their environmental consequences. This process entails the presence of bacteria that harbor specific enzymes or metabolic pathways capable of degrading antibiotic molecules, thereby utilizing the resultant degradation products for their growth and proliferation. For example, *Pseudomonas fluorescens* has been documented to utilize oxytetracycline as its exclusive carbon source, fully mineralizing it into carbon dioxide and water (Egorov et al., 2018). Comparably, *Pseudomonas putida* can degrade penicillin-G through hydrolysis, forming penicilloic acid and phenylacetic acid. This microbe subsequently employs phenylacetic acid as a carbon source (Al-Ahmad et al., 1999). This mechanism is similarly relevant to a range of pollutants, including petroleum hydrocarbons, where bacteria like *Pseudomonas putida* employ these compounds as carbon sources for their growth, illustrating a well-documented bioremediation strategy (Cui et al., 2020). The application of this nutrient presents significant benefits for bioremediation, as it leverages the natural metabolic processes of bacteria to restore contaminated environments, particularly those impacted by antibiotic-laden wastewater or soil. By meticulously selecting or engineering bacterial strains that exhibit distinct degradation pathways, it is possible to formulate bioremediation strategies specifically designed to target particular pollutants. For example, studies have effectively isolated bacterial strains capable of degrading sulfonamides, fluoroquinolones, and other antibiotics, using these compounds as carbon sources, which enhances remediation efforts (Fu et al., 2022; Wang et al., 2023; Zhang M. et al., 2023; Rodrigues et al., 2025; Zhu et al., 2025) (Table 3).

Nevertheless, one must consider the significant challenges, including the specificity exhibited by various bacterial strains toward certain antibiotics and the potential to spread ARGs. It is essential to recognize that not all antibiotics function as suitable carbon sources. The effectiveness of this application depends on the distinct chemical composition of the antibiotic involved, alongside the specific bacterial strain under investigation. Moreover, bacteria that possess resistance mechanisms, such as the production of beta-lactamases, can transfer resistance genes to other microorganisms in their surroundings, thus posing considerable threats to ecological systems (Markley and Wencewicz, 2018). Ongoing research endeavors are concentrated on enhancing this methodology, exploring the domain of genetic engineering to augment degradation efficiency while considering the possible risks associated with resistance.

##### Biofilm production

Biofilms, complex communities of microorganisms, play a significant role in bioremediation by degrading a wide range of environmental contaminants, such as petroleum hydrocarbons and heavy metals, due to their resilience to harsh conditions like toxic chemicals, desiccation, and UV radiation, as supported by studies like Ian W. Sutherland’s work on biofilm exopolysaccharides (Sutherland, 2001). Their extracellular matrix contains functional groups that bind and immobilize hydrophobic compounds, facilitating degradation, as shown in the study by Mangwani et al. (2016), making them suitable for cleaning up contaminated sites, as noted by Das and Dash (2014). Natural attenuation, where indigenous microbes degrade contaminants without intervention, can be slow, especially in newly contaminated sites (Sara, 2003), but can be accelerated through biostimulation, adding nutrients like nitrogen or carbon, and bioaugmentation, introducing specific degradative microorganisms (Tyagi et al., 2011; Sonawane et al., 2022). Additionally, changes in biofilm physiology and morphology can detect contaminants, such as heavy metals, microplastics, and other pollutants in water (Syed et al., 2021; Luo et al., 2022), serving as indicators, offering a promising approach for bioremediation with ongoing research focused on optimizing these processes.

##### Consortia mechanisms

**Figure 4:**
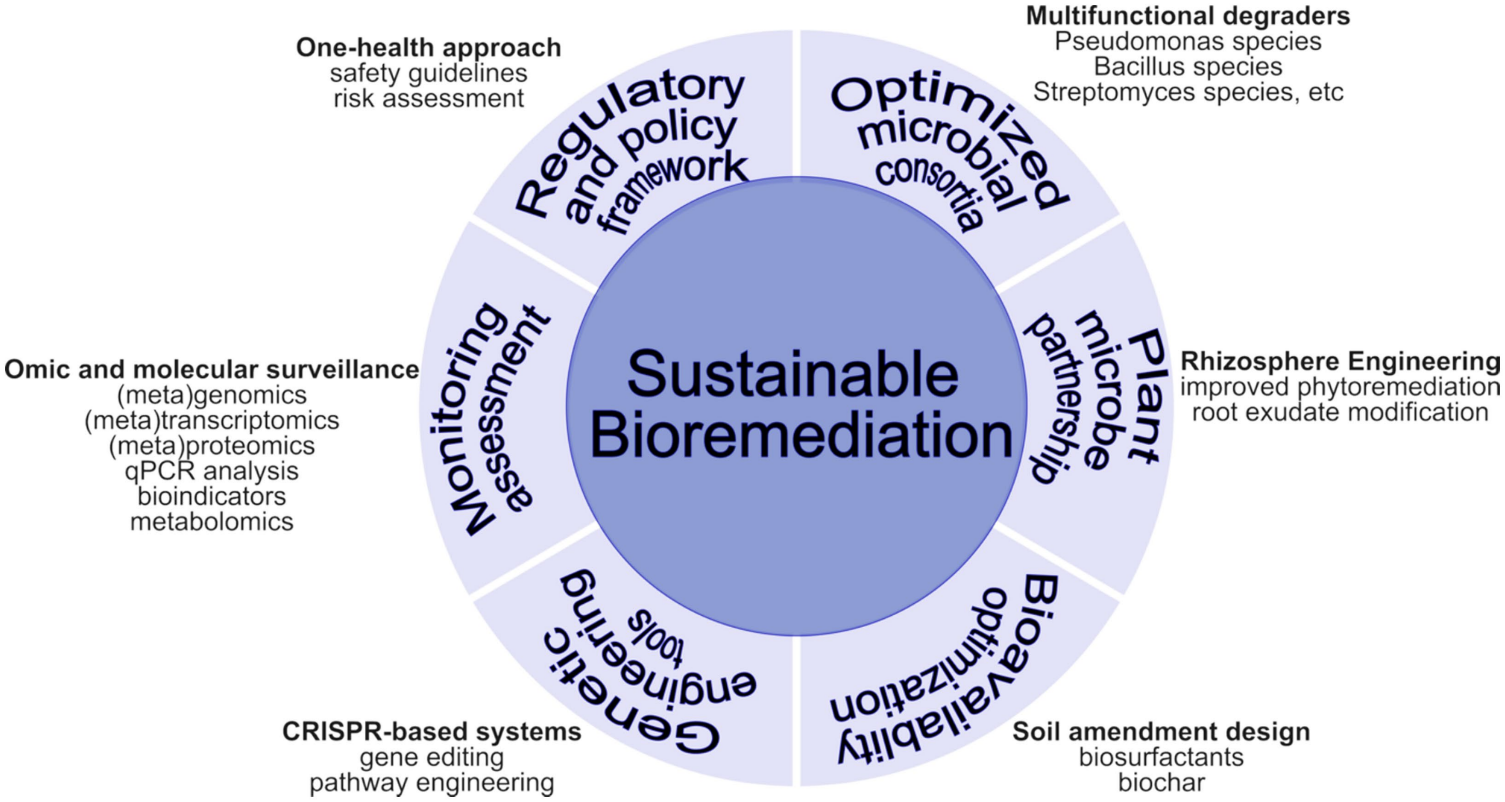
Integrated approach to antibiotic bioremediation in agricultural soils.

Microbial consortia, defined as cooperative assemblies of microorganisms, play a crucial role in bioremediation by collaboratively degrading environmental pollutants that individual strains may struggle to address (Cieplik et al., 2019) (Figure 4). Their effectiveness is attributed to their ability to participate in diverse metabolic processes, which enables them to adjust to complex combinations of pollutants and varying environmental circumstances, as evidenced by studies showing enhanced degradation rates in areas impacted by oil contamination (Valentine et al., 2010). For instance, naturally occurring bacterial consortia, exemplified by *Alcanivorax borkumensis*, have been instrumental in the bioremediation efforts following the Deepwater Horizon oil spill in 2010, resulting in a notable decrease in the environmental repercussions of crude oil. The collaborative endeavors evident within these consortia manifest in diverse manners, enhancing their potential for bioremediation. Some microorganisms are recognized for their ability to synthesize biosurfactants, such as rhamnolipids, which emulsify hydrophobic pollutants like oil, thus improving their bioavailability for degradation by other constituents of the microbial community (Zeng et al., 2018). Sequential degradation serves as a crucial mechanism within microbial ecology, where an initial microorganism commences the breakdown of a pollutant, thereby enabling a subsequent organism to complete the degradation process. An illustrative example can be found in the bioremediation of polychlorinated biphenyls, where anaerobic bacteria first engage in dechlorinating these compounds, after which aerobic bacteria mineralize the resulting intermediates (Lin et al., 2024). This collaborative methodology enables a more comprehensive dissection of complex pollutants, surpassing the efficacy of singular strains.

Engineered microbial consortia are presently being developed to target pollutants precisely, thus offering tailored bioremediation solutions. For example, studies have demonstrated that a consortium of *Pseudomonas putida* and *Acinetobacter* sp. achieved enhanced phenol degradation rates compared to either strain alone, highlighting the benefits of combining complementary metabolic pathways (Xu et al., 2024). These methodologies present considerable promise for addressing emerging contaminants, such as pharmaceuticals and pesticides, especially in cases where natural consortia may demonstrate insufficient activity. Despite these advantages, challenges remain in applying microbial consortia for bioremediation efforts. It is essential to ensure the stability of a consortium when faced with fluctuating environmental conditions, as competition among its members or changes in the environment can greatly compromise cooperative interactions (Bernal and Llamas, 2012). Moreover, it is imperative to contemplate the possibility of propagating antibiotic resistance genes or other unfavorable traits within the consortium, which could result in ecological consequences (Piazza et al., 2019). Such challenges necessitate careful supervision and strategic administration to ensure effectiveness and safety. In considering the trajectory of future advancements, it is evident that the domains of synthetic biology and genomics are poised to revolutionize the approaches utilized in the design and optimization of microbial consortia. Researchers can develop more efficient and manageable bioremediation strategies by engineering microbes to exhibit specific degradation pathways or enhance their cooperative behaviors through genetic modifications (Singh et al., 2011). The engineering of synthetic consortia is designed to degrade a broader spectrum of pollutants, thus offering scalable methodologies for environmental remediation.

### Molecular methods versus established bioremediation technologies

Targeted molecular tools such as CRISPR-Cas systems delivered by bacteriophages or conjugative plasmids can selectively remove ARGs or be used to control ARG-carrying hosts (e.g., plasmid curing or sequence-specific killing), offering precision with minimal collateral damage to non-targets when delivery succeeds. Proof-of-concept studies show >99.9% elimination of targeted resistant *E. coli in vivo* using conjugative CRISPR-Cas9 in the gut microbiota (Neil et al., 2021), phage-delivered CRISPR that degrades plasmid-borne ARGs without killing hosts (Tao et al., 2022), CRISPR systems (e.g., VADER) that degrade ARGs in wastewater matrices (Li X. et al., 2023), and engineered anti-biofilm phages that outperform wild-type phages *in vitro* and reduce intestinal *E. coli* in animals (Gencay et al., 2024). However, soil deployment remains nascent: reviews emphasize delivery barriers in complex matrices, risks of escape mutants/off-target effects, and biosafety/regulatory uncertainties, arguing that near-term use is likeliest in contained nodes (e.g., digesters, WWTPs) rather than open soils (Mayorga-Ramos et al., 2023).

By contrast, established feasible treatments are field-ready but less specific. Manure composting often reduces ARG loads and resistome risk relative to stockpiling, though marker-specific rebounds can occur; performance depends on temperature profiles and process control (Keenum et al., 2021; Sun et al., 2024; Wang G. et al., 2024). Biochar amendments can sorb antibiotics, shift microbial communities, and lower ARG abundances in soil-crop systems, but long-term persistence and trade-offs remain under study (Choi et al., 2024; Li H. et al., 2024). Advanced oxidation processes (AOPs) such as ozonation, UV/H₂O₂, and photocatalysis efficiently remove antibiotics in water (Cuerda-Correa et al., 2020; Zhang Y. et al., 2022; Pastor-Lopez et al., 2024) and, in some cases, reduce ARB/ARGs, making them suitable upstream of land application; however, limitations include energy cost and by-product control, and they are not directly applicable in soils. Overall, gene-editing tools are promising and precise but currently best suited to contained treatment applications; composting/biochar/AOPs remain deployable at scale for agricultural settings, with method choice governed by matrix (manure, soil, wastewater), cost, and risk targets (Mayorga-Ramos et al., 2023).

## Regulatory considerations and practical challenges on the application of beneficial microbes in bioremediation

Bacterial bioremediation can help clean antibiotic-polluted farm soils, but it has significant regulatory and practical challenges. Tight rules on genetically modified organisms (GMOs), different ways to manage antibiotics, and ecological uncertainties make deployment tricky. Practical challenges like microbial survival, cost-effectiveness, and soil variability limit scalability. Hence, we examine barriers, highlighting their impact on microbial solutions and the need for integrated strategies to realize the bioremediation potential of beneficial bacteria.

Regulatory frameworks for antibiotic use and microbial bioremediation vary widely from country to country and region to region, creating uneven landscapes for implementation. In the European Union (EU), strict regulations, such as Regulation (EU) 2019/6, have banned antibiotic growth promoters in livestock since 2006 (Topp et al., 2018; Mdegela et al., 2021; Farrukh et al., 2025). However, therapeutic use remains high, with 131 mg/kg of poultry biomass treated annually, maintaining ARG hotspots (Checcucci et al., 2020). In regions like South Asia and parts of Africa, lax oversight allows subtherapeutic dosing, with up to 70% of veterinary antibiotics excreted unchanged, boosting soil ARG loads by 10^3^ copies/g (Muhammad et al., 2020). This disparity hampers consistent bioremediation strategies, as bacterial strains face different antibiotic pressures in their immediate environments.

Also, the regulations surrounding genetically modified organisms impose additional constraints on using CRISPR-edited bacterial strains. The European Union’s Directive 2001/18/EC categorizes CRISPR-modified strains as GMOs (Okoli et al., 2022), necessitating extensive risk assessments and public approval processes. This affects the benefits of this technology. For example, the field trials of *Pseudomonas* with a silenced *intI1* gene were postponed, notwithstanding the success observed in laboratory settings (Ferreira et al., 2023). On the other hand, the USDA’s streamlined process under the “SECURE Rule” permits certain gene-edited microbes to bypass GMO labeling in the United States, provided that no foreign DNA is present. This facilitates pilot tests of *Bacillus subtilis* that have been enhanced with the *tetX* gene (Kim et al., 2023). In developing nations, it is evident that regulatory frameworks frequently exhibit significant deficiencies, thereby posing a risk of uncontrolled release and subsequent ecological damage. Achieving coherence among these policies has the potential to establish a standardized approach to safe deployment, however, one must acknowledge the ongoing political and economic obstacles.

In addition, the cost of production is a major barrier to widespread adoption. Smallholder farmers, common in developing regions, often lack access to expensive technologies needed for production. Around 80% rely on manure without remediation (Muhammad et al., 2020). Scaling requires reducing costs, such as optimizing bioreactor yields (10^1^⁰ CFU/mL) or using local isolates, but infrastructure gaps remain. Soil variability makes scaling even more challenging. For example, *sadA*-expressing *Pseudomonas* degraded SMX 70% faster in acidic loams (pH 5.5, 5% organic matter) than in neutral clays (pH 7, 10% organic matter) due to sorption issues (Rodríguez-López et al., 2024). Also, in another study, field trials across ten sites showed consortia efficacy ranging from 20 to 80% in ARG reduction, linked to specific microbial interactions in the soil (Wang S. et al., 2024). Hence, tailoring strains to local conditions through omics-guided selection could enhance results, but this requires extensive regional data and adaptive formulations, which are expensive.

Finally, addressing these challenges needs integrated strategies. These strategies can transform lab research into practical applications, but coordinated efforts across microbiology, policy, and agriculture are crucial.

## Conclusion and future recommendations

This review has comprehensively examined the challenges of antibiotic contamination in agricultural soils and the promising potential of beneficial bacteria in bioremediation. By detailing the complex sources of antibiotic pollution, the influence of soil parameters on antibiotic bioavailability, and the mechanisms by which microbial consortia can degrade antibiotics (e.g., enzymatic biodegradation, biofilm formation, and cooperative metabolism), the manuscript demonstrates how integrating molecular insights with practical remediation strategies can address both ecological and public health concerns. The discussion underscores that despite the encouraging laboratory-scale findings, challenges such as scalability, cost-effectiveness, and potential environmental risks associated with deploying engineered microbes persist.

The symbiotic association between plants, soil, and beneficial microbes has been shown to play a pivotal role in bioremediation and plant health improvement. However, a lot of information is still missing to make more positive developments from these interactions. A good bioremediation strategy involves a well-designed and targeted approach involving various approaches to achieve sustainable bioremediation (Figure 4). For full acceptance of beneficial microbes in bioremediation and sustainable agriculture, more insight is needed into the comprehensive tripartite interactions between plants, microbes, and their environment. Knowledge of microbial functions for bioremediation purposes is also very critical. Identification of candidate genes involved in antibiotics degradation and their characterization are necessary to allow for possible manipulation and editing of these genes for improved bioremediation potential and plant health promotion. Several regulatory mechanisms should also be looked into regarding genetically modified organisms for the safety of potential bioremediation potentials in the environment, since they will be deployed in the field to ascertain their potential fully.

At the center is the core objective of sustainable antibiotic bioremediation. Surrounding this are key components: (1) Optimized Microbial Consortia—featuring multi-functional degrader bacteria engineered for complementary metabolic capabilities, (2) Plant-Microbe Partnership Systems—emphasizing rhizosphere engineering to enhance plant-microbial synergies in the root zone, (3) Bioavailability Optimization Strategies—focusing on soil amendment design that considers pH, clay content, organic matter, and moisture effects, (4) Monitoring and Assessment—employing metagenomic surveillance to track antibiotic resistance genes and remediation progress, (5) Genetic Engineering Tools—utilizing CRISPR-based systems for enhanced degradation capabilities, and (6) Regulatory and Policy Framework—implementing a One Health approach aligned with Sustainable Development Goals 2 (Zero Hunger), 3 (Good Health and Well-being), and 15 (Life on Land).

Looking forward, future research should prioritize a few key areas. First, experimental approaches must be refined to quantify better how specific soil properties (pH, organic matter, texture) modulate antibiotic persistence and degradation rates, leveraging advanced metagenomic and metabolomic tools. Second, further characterization and optimization of microbial consortia, potentially through synthetic biology and gene editing, will be crucial in enhancing bioremediation efficacy while ensuring biosafety. Third, extensive field trials under diverse agricultural conditions are needed to validate laboratory findings and to tailor remediation strategies to local contexts. Finally, harmonizing regulatory frameworks internationally, supported by comprehensive risk assessments and stakeholder engagement, is essential to safely advance the application of natural and engineered microbial remediation methods (Figure 4). These integrated strategies can significantly contribute to sustainable agriculture and environmental protection under a One Health framework by bridging molecular-level research with policy and field implementation.

To chart a path forward, we propose key unanswered questions that can bridge laboratory insights and field realities. These targeted research priorities will help focus efforts on linking soil properties, microbial dynamics, and stakeholder needs for effective, scalable bioremediation.

1. How do combined soil parameters, i.e., pH, organic matter, and texture, interact to modulate *in situ* degradation rates of distinct antibiotic classes?
2. Which native microbial consortia optimize the co-degradation of multi-class antibiotic occurrences under field conditions?
3. What is the extent of horizontal gene transfer of ARGs during bioaugmentation with introduced versus indigenous bacteria?
4. How does plant–microbe–soil feedback influence the long-term stability of bioremediation efficacy and crop uptake of antibiotic residues?
5. What socio-economic factors most strongly predict smallholder farmer adoption of microbial bioremediation technologies?
6. Can metagenome-informed predictive models accurately forecast bioremediation outcomes across diverse agroecosystems?

## References

[ref1] Ahmed D. A. E.-A. Slima D. F. Al-Yasi H. M. Hassan L. M. Galal T. M. (2023). Risk assessment of trace metals in *Solanum lycopersicum* L. (tomato) grown under wastewater irrigation conditions. Environ. Sci. Pollut. Res. 30, 42255–42266. doi: 10.1007/s11356-023-25157-8, PMID: 36645601 PMC10067660

[ref2] Al-Ahmad A. Daschner F. D. Kümmerer K. (1999). Biodegradability of cefotiam, ciprofloxacin, meropenem, penicillin G, and sulfamethoxazole and inhibition of waste water bacteria. Arch. Environ. Contam. Toxicol. 37, 158–163. doi: 10.1007/s002449900501, PMID: 10398765

[ref3] Azanu D. Mortey C. Darko G. Weisser J. J. Styrishave B. Abaidoo R. C. (2016). Uptake of antibiotics from irrigation water by plants. Chemosphere 157, 107–114. doi: 10.1016/j.chemosphere.2016.05.035, PMID: 27213239

[ref4] Banerjee S. Van Der Heijden M. G. A. (2023). Soil microbiomes and one health. Nat. Rev. Microbiol. 21, 6–20. doi: 10.1038/s41579-022-00779-w, PMID: 35999468

[ref5] Batuman O. Britt-Ugartemendia K. Kunwar S. Yilmaz S. Fessler L. Redondo A.. (2024). The use and impact of antibiotics in plant agriculture: A review. Phytopathology 114, 885–909. doi: 10.1094/PHYTO-10-23-0357-IA, PMID: 38478738

[ref6] Bengtsson-Palme J. Larsson D. G. J. (2016). Concentrations of antibiotics predicted to select for resistant bacteria: proposed limits for environmental regulation. Environ. Int. 86, 140–149. doi: 10.1016/j.envint.2015.10.015, PMID: 26590482

[ref7] Bernal P. Llamas M. A. (2012). Promising biotechnological applications of antibiofilm exopolysaccharides. Microb. Biotechnol. 5, 670–673. doi: 10.1111/j.1751-7915.2012.00359.x, PMID: 22905927 PMC3815889

[ref8] Bhandari S. Poudel D. K. Marahatha R. Dawadi S. Khadayat K. Phuyal S.. (2021). Microbial enzymes used in bioremediation. J. Chem. 2021, 1–17. doi: 10.1155/2021/8849512, PMID: 40842940

[ref9] Bilal M. Mehmood S. Rasheed T. Iqbal H. M. N. (2020). Antibiotics traces in the aquatic environment: persistence and adverse environmental impact. Curr. Opin. Environ. Sci. Health 13, 68–74. doi: 10.1016/j.coesh.2019.11.005

[ref10] Billet L. Pesce S. Rouard N. Spor A. Paris L. Leremboure M.. (2021). Antibiotrophy: key function for antibiotic-resistant Bacteria to colonize soils—case of sulfamethazine-degrading *Microbacterium* sp. C448. Front. Microbiol. 12:643087. doi: 10.3389/fmicb.2021.643087, PMID: 33841365 PMC8032547

[ref11] Bougnom B. P. Thiele-Bruhn S. Ricci V. Zongo C. Piddock L. J. V. (2020). Raw wastewater irrigation for urban agriculture in three African cities increases the abundance of transferable antibiotic resistance genes in soil, including those encoding extended spectrum beta-lactamases (ESBLs). Sci. Total Environ. 698:134201. doi: 10.1016/j.scitotenv.2019.134201 31505362

[ref12] Bravo G. Vega-Celedón P. Gentina J. C. Seeger M. (2020). Bioremediation by *Cupriavidus metallidurans* strain MSR33 of mercury-polluted agricultural soil in a rotary drum bioreactor and its effects on nitrogen cycle microorganisms. Microorganisms 8:1952. doi: 10.3390/microorganisms8121952, PMID: 33316980 PMC7763483

[ref13] Bush K. (2010). Bench-to-bedside review: the role of beta-lactamases in antibiotic-resistant Gram-negative infections. Crit. Care 14:224. doi: 10.1186/cc8892, PMID: 20594363 PMC2911681

[ref14] Buta M. Hubeny J. Zielinski W. Harnisz M. Korzeniewska E. (2021). Sewage sludge in agriculture - the effects of selected chemical pollutants and emerging genetic resistance determinants on the quality of soil and crops - a review. Ecotoxicol. Environ. Saf. 214:112070. doi: 10.1016/j.ecoenv.2021.112070, PMID: 33652361

[ref15] Caneschi A. Bardhi A. Barbarossa A. Zaghini A. (2023). The use of antibiotics and antimicrobial resistance in veterinary medicine, a complex phenomenon: A narrative review. Antibiotics (Basel) 12:487. doi: 10.3390/antibiotics12030487, PMID: 36978354 PMC10044628

[ref16] Checcucci A. Trevisi P. Luise D. Modesto M. Blasioli S. Braschi I.. (2020). Exploring the animal waste Resistome: the spread of antimicrobial resistance genes through the use of livestock manure. Front. Microbiol. 11:1416. doi: 10.3389/fmicb.2020.01416, PMID: 32793126 PMC7387501

[ref17] Chen Q. L. Cui H. L. Su J. Q. Penuelas J. Zhu Y. G. (2019). Antibiotic Resistomes in plant microbiomes. Trends Plant Sci. 24, 530–541. doi: 10.1016/j.tplants.2019.02.010, PMID: 30890301

[ref18] Chen B. He R. Yuan K. Chen E. Lin L. Chen X.. (2017). Polycyclic aromatic hydrocarbons (PAHs) enriching antibiotic resistance genes (ARGs) in the soils. Environ. Pollut. 220, 1005–1013. doi: 10.1016/j.envpol.2016.11.047, PMID: 27876418

[ref19] Chen Z. Liu X. Chen L. Han Y. Shen Y. Chen B.. (2023). Deglycosylation inactivation initiated by a novel periplasmic dehydrogenase complex provides a novel strategy for eliminating the recalcitrant antibiotic kanamycin. Environ. Sci. Technol. 57, 4298–4307. doi: 10.1021/acs.est.2c09565, PMID: 36857046

[ref20] Chen X. Song Y. Ling C. Shen Y. Zhan X. Xing B. (2024). Fate of emerging antibiotics in soil-plant systems: A case on fluoroquinolones. Sci. Total Environ. 951:175487. doi: 10.1016/j.scitotenv.2024.175487, PMID: 39153616

[ref21] Choi G. Brady J. A. Obayomi O. Green E. Leija C. Sefcik K.. (2024). Wood- and manure-derived biochars reduce antibiotic residues and shift antibiotic resistance genes and microbial communities in manure applied forage–soil systems. Agronomy 14:2100. doi: 10.3390/agronomy14092100

[ref22] Christou A. Aguera A. Bayona J. M. Cytryn E. Fotopoulos V. Lambropoulou D.. (2017). The potential implications of reclaimed wastewater reuse for irrigation on the agricultural environment: the knowns and unknowns of the fate of antibiotics and antibiotic resistant bacteria and resistance genes - A review. Water Res. 123, 448–467. doi: 10.1016/j.watres.2017.07.004, PMID: 28689129

[ref23] Cieplik F. Jakubovics N. S. Buchalla W. Maisch T. Hellwig E. Al-Ahmad A. (2019). Resistance toward chlorhexidine in Oral Bacteria - is there cause for concern? Front. Microbiol. 10:587. doi: 10.3389/fmicb.2019.00587, PMID: 30967854 PMC6439480

[ref24] Cuerda-Correa E. M. Alexandre-Franco M. F. Fernández-González C. (2020). Advanced oxidation processes for the removal of antibiotics from water. An overview. Water 12:102. doi: 10.3390/w12010102

[ref25] Cui G. Li X. D. Yang M. Ding S. Li Q. K. Wang Y.. (2020). Insight into the mechanisms of denitrification and sulfate reduction coexistence in cascade reservoirs of the Jialing River: evidence from a multi-isotope approach. Sci. Total Environ. 749:141682. doi: 10.1016/j.scitotenv.2020.141682, PMID: 33370886

[ref26] Cui S. Qi Y. Zhu Q. Wang C. Sun H. (2023). A review of the influence of soil minerals and organic matter on the migration and transformation of sulfonamides. Sci. Total Environ. 861:160584. doi: 10.1016/j.scitotenv.2022.160584, PMID: 36455724

[ref27] Cycon M. Mrozik A. Piotrowska-Seget Z. (2019). Antibiotics in the soil environment-degradation and their impact on microbial activity and diversity. Front. Microbiol. 10:338. doi: 10.3389/fmicb.2019.00338, PMID: 30906284 PMC6418018

[ref28] D'angelo E. M. (2023). Diversity of virulence and antibiotic resistance genes expressed in class A biosolids and biosolids-amended soil as revealed by metatranscriptomic analysis. Lett. Appl. Microbiol. 76:ovad097. doi: 10.1093/lambio/ovad097, PMID: 37596067

[ref29] Danilova N. Galieva G. Kuryntseva P. Selivanovskaya S. Galitskaya P. (2023). Influence of the antibiotic Oxytetracycline on the morphometric characteristics and endophytic bacterial Community of Lettuce (*Lactuca sativa* L.). Microorganisms 11:2828. doi: 10.3390/microorganisms11122828, PMID: 38137972 PMC10746115

[ref30] Danner M. C. Robertson A. Behrends V. Reiss J. (2019). Antibiotic pollution in surface fresh waters: occurrence and effects. Sci. Total Environ. 664, 793–804. doi: 10.1016/j.scitotenv.2019.01.406, PMID: 30763859

[ref31] Das S. Dash H. R. (2014). “Microbial Bioremediation” in Microbial biodegradation and bioremediation. ed. Das S. (Oxford: Elsevier), 1–21.

[ref32] De Farias B. O. Saggioro E. M. Montenegro K. S. Magaldi M. Santos H. S. O. Goncalves-Brito A. S.. (2024). Metagenomic insights into plasmid-mediated antimicrobial resistance in poultry slaughterhouse wastewater: antibiotics occurrence and genetic markers. Environ. Sci. Pollut. Res. Int. 31, 60880–60894. doi: 10.1007/s11356-024-35287-2, PMID: 39395082

[ref33] Deng J. Zhang W. Zhang L. Qin C. Wang H. Ling W. (2024). Micro-interfacial behavior of antibiotic-resistant bacteria and antibiotic resistance genes in the soil environment: A review. Environ. Int. 191:108972. doi: 10.1016/j.envint.2024.108972, PMID: 39180776

[ref34] Ding C. He J. (2010). Effect of antibiotics in the environment on microbial populations. Appl. Microbiol. Biotechnol. 87, 925–941. doi: 10.1007/s00253-010-2649-5, PMID: 20508933

[ref35] Ding M. Ye Z. Liu L. Wang W. Chen Q. Zhang F.. (2022). Subinhibitory antibiotic concentrations promote the horizontal transfer of plasmid-borne resistance genes from *Klebsiellae pneumoniae* to *Escherichia coli*. Front. Microbiol. 13:1017092. doi: 10.3389/fmicb.2022.1017092, PMID: 36419429 PMC9678054

[ref36] Dong J. Xie H. Feng R. Lai X. Duan H. Xu L.. (2021). Transport and fate of antibiotics in a typical aqua-agricultural catchment explained by rainfall events: implications for catchment management. J. Environ. Manag. 293:112953. doi: 10.1016/j.jenvman.2021.112953, PMID: 34102496

[ref37] Du L. F. Liu W. K. (2012). Occurrence, fate, and ecotoxicity of antibiotics in agro-ecosystems. A review. Agron. Sustain. Dev. 32, 309–327. doi: 10.1007/s13593-011-0062-9

[ref38] Egorov A. M. Ulyashova M. M. Rubtsova M. Y. (2018). Bacterial enzymes and antibiotic resistance. Acta Nat. 10, 33–48. doi: 10.32607/20758251-2018-10-4-33-48, PMID: 30713760 PMC6351036

[ref39] Elarabi N. I. Halema A. A. Abdelhadi A. A. Henawy A. R. Samir O. Abdelhaleem H. A. R. (2023). Draft genome of *Raoultella planticola*, a high lead resistance bacterium from industrial wastewater. AMB Express 13:14. doi: 10.1186/s13568-023-01519-w, PMID: 36715862 PMC9885416

[ref40] Elder F. C. T. O'neill A. J. Collins L. M. Carter L. J. (2023). A framework to assess the terrestrial risk of antibiotic resistance from antibiotics in slurry or manure amended soils. Environ. Sci. Adv. 2, 780–794. doi: 10.1039/D2VA00306F

[ref41] Ezugworie F. N. Igbokwe V. C. Onwosi C. O. (2021). Proliferation of antibiotic-resistant microorganisms and associated genes during composting: an overview of the potential impacts on public health, management and future. Sci. Total Environ. 784:147191. doi: 10.1016/j.scitotenv.2021.147191, PMID: 33905939

[ref42] Fang H. Oberoi A. S. He Z. Khanal S. K. Lu H. (2021). Ciprofloxacin-degrading *Paraclostridium* sp. isolated from sulfate-reducing bacteria-enriched sludge: optimization and mechanism. Water Res. 191:116808. doi: 10.1016/j.watres.2021.116808, PMID: 33454651

[ref43] Farrukh M. Munawar A. Nawaz Z. Hussain N. Hafeez A. B. Szweda P. (2025). Antibiotic resistance and preventive strategies in foodborne pathogenic bacteria: a comprehensive review. Food Sci. Biotechnol. 34, 2101–2129. doi: 10.1007/s10068-024-01767-x, PMID: 40351726 PMC12064539

[ref44] Fatoba D. O. Abia A. L. K. Amoako D. G. Essack S. Y. (2021). Rethinking manure application: increase in multidrug-resistant Enterococcus spp. in agricultural soil following chicken litter application. Microorganisms 9:885. doi: 10.3390/microorganisms9050885, PMID: 33919134 PMC8170873

[ref45] Ferreira C. Otani S. Aarestrup F. M. Manaia C. M. (2023). Quantitative PCR versus metagenomics for monitoring antibiotic resistance genes: balancing high sensitivity and broad coverage. FEMS Microbes 4:xtad008. doi: 10.1093/femsmc/xtad008, PMID: 37333442 PMC10117749

[ref46] Forsberg K. J. Reyes A. Wang B. Selleck E. M. Sommer M. O. Dantas G. (2012). The shared antibiotic resistome of soil bacteria and human pathogens. Science 337, 1107–1111. doi: 10.1126/science.1220761, PMID: 22936781 PMC4070369

[ref47] Freitag C. Michael G. B. Li J. Kadlec K. Wang Y. Hassel M.. (2018). Occurrence and characterisation of ESBL-encoding plasmids among *Escherichia coli* isolates from fresh vegetables. Vet. Microbiol. 219, 63–69. doi: 10.1016/j.vetmic.2018.03.028, PMID: 29778206

[ref48] Frey L. Tanunchai B. Glaser B. (2022). Antibiotics residues in pig slurry and manure and its environmental contamination potential. A meta-analysis. Agron. Sustain. Dev. 42:31. doi: 10.1007/s13593-022-00762-y

[ref49] Fu R. Li X. Zhao Y. Pu Q. Li Y. Gu W. (2022). Efficient and synergistic degradation of fluoroquinolones by bacteria and microalgae: design of environmentally friendly substitutes, risk regulation and mechanism analysis. J. Hazard. Mater. 437:129384. doi: 10.1016/j.jhazmat.2022.129384, PMID: 35897172

[ref50] Gasparrini A. J. Markley J. L. Kumar H. Wang B. Fang L. Irum S.. (2020). Tetracycline-inactivating enzymes from environmental, human commensal, and pathogenic bacteria cause broad-spectrum tetracycline resistance. Commun. Biol. 3:241. doi: 10.1038/s42003-020-0966-5 32415166 PMC7229144

[ref51] Gatica J. Cytryn E. (2013). Impact of treated wastewater irrigation on antibiotic resistance in the soil microbiome. Environ. Sci. Pollut. Res. Int. 20, 3529–3538. doi: 10.1007/s11356-013-1505-4, PMID: 23378260 PMC3646162

[ref52] Gencay Y. E. Jasinskytė D. Robert C. Semsey S. Martínez V. Petersen A. Ø.. (2024). Engineered phage with antibacterial CRISPR–Cas selectively reduce *E. coli* burden in mice. Nat. Biotechnol. 42, 265–274. doi: 10.1038/s41587-023-01759-y, PMID: 37142704 PMC10869271

[ref53] Geng J. Liu X. Wang J. Li S. (2022). Accumulation and risk assessment of antibiotics in edible plants grown in contaminated farmlands: A review. Sci. Total Environ. 853:158616. doi: 10.1016/j.scitotenv.2022.158616, PMID: 36089029

[ref54] Ghirardini A. Grillini V. Verlicchi P. (2020). A review of the occurrence of selected micropollutants and microorganisms in different raw and treated manure - environmental risk due to antibiotics after application to soil. Sci. Total Environ. 707:136118. doi: 10.1016/j.scitotenv.2019.136118, PMID: 31881518

[ref55] Gonzalez Ronquillo M. Angeles Hernandez J. C. (2017). Antibiotic and synthetic growth promoters in animal diets: review of impact and analytical methods. Food Control 72, 255–267. doi: 10.1016/j.foodcont.2016.03.001

[ref56] Gromala M. Neufeld J. D. Mcconkey B. J. (2021). Monitoring microbial populations and antibiotic resistance gene enrichment associated with Arctic waste stabilization ponds. Appl. Environ. Microbiol. 87, e02914–e02920. doi: 10.1128/AEM.02914-20 33452030 PMC8091602

[ref57] Gu J. Y. Chen C. Y. Huang X. Y. Mo J. C. Xie Q. L. Zeng Q. Y. (2021). Occurrence and risk assessment of tetracycline antibiotics in soils and vegetables from vegetable fields in Pearl River Delta, South China. Sci. Total Environ. 776:145959. doi: 10.1016/j.scitotenv.2021.145959

[ref58] Guo Y. (2021). Removal ability of antibiotic resistant Bacteria (arb) and antibiotic resistance genes (Args) by membrane filtration process. IOP conference series: earth and environmental science 801:012004. doi: 10.1088/1755-1315/801/1/012004

[ref59] Guo J. Li J. Chen H. Bond P. L. Yuan Z. (2017). Metagenomic analysis reveals wastewater treatment plants as hotspots of antibiotic resistance genes and mobile genetic elements. Water Res. 123, 468–478. doi: 10.1016/j.watres.2017.07.002, PMID: 28689130

[ref60] Gwenzi W. Shamsizadeh Z. Gholipour S. Nikaeen M. (2022). The air-borne antibiotic resistome: occurrence, health risks, and future directions. Sci. Total Environ. 804:150154. doi: 10.1016/j.scitotenv.2021.150154, PMID: 34798728

[ref61] Gworek B. Kijenska M. Wrzosek J. Graniewska M. (2021). Pharmaceuticals in the soil and plant environment: a review. Water Air Soil Pollut. 232:145. doi: 10.1007/s11270-020-04954-8

[ref62] Haider M. I. S. Liu G. Yousaf B. Arif M. Aziz K. Ashraf A.. (2024). Synergistic interactions and reaction mechanisms of biochar surface functionalities in antibiotics removal from industrial wastewater. Environ. Pollut. 356:124365. doi: 10.1016/j.envpol.2024.124365, PMID: 38871166

[ref63] Han B. Ma L. Yu Q. Yang J. Su W. Hilal M. G.. (2022). The source, fate and prospect of antibiotic resistance genes in soil: A review. Front. Microbiol. 13:976657. doi: 10.3389/fmicb.2022.976657, PMID: 36212863 PMC9539525

[ref64] He J. Z. Yan Z. Z. Chen Q. L. (2020). Transmission of antibiotic resistance genes in agroecosystems: an overview. Front. Agric. Sci. Eng. 7, 329–332. doi: 10.15302/J-FASE-2020333

[ref65] Hilaire S. S. Chen C. Pan Z. Radolinski J. Stewart R. D. Maguire R. O.. (2022). Subsurface manure injection reduces surface transport of antibiotic resistance genes but may create antibiotic resistance hotspots in soils. Environ. Sci. Technol. 56, 14972–14981. doi: 10.1021/acs.est.2c00981, PMID: 35839145

[ref66] Hong X. Zhao Y. Zhuang R. Liu J. Guo G. Chen J.. (2020). Bioremediation of tetracycline antibiotics-contaminated soil by bioaugmentation. RSC Adv. 10, 33086–33102. doi: 10.1039/D0RA04705H, PMID: 35694106 PMC9122622

[ref67] Hutchings M. I. Truman A. W. Wilkinson B. (2019). Antibiotics: past, present and future. Curr. Opin. Microbiol. 51, 72–80. doi: 10.1016/j.mib.2019.10.008, PMID: 31733401

[ref68] Iwu C. D. Korsten L. Okoh A. I. (2020). The incidence of antibiotic resistance within and beyond the agricultural ecosystem: A concern for public health. Microbiology 9:e1035. doi: 10.1002/mbo3.1035, PMID: 32710495 PMC7520999

[ref69] Jauregi L. Epelde L. Gonzalez A. Lavin J. L. Garbisu C. (2021). Reduction of the resistome risk from cow slurry and manure microbiomes to soil and vegetable microbiomes. Environ. Microbiol. 23, 7643–7660. doi: 10.1111/1462-2920.15842, PMID: 34792274

[ref70] Jia Y. Y. Ou Y. Y. Khanal S. K. Sun L. P. Shu W. S. Lu H. (2024). Biochar-based strategies for antibiotics removal: mechanisms, factors, and application. Acs ES&T Eng. 4, 1256–1274. doi: 10.1021/acsestengg.3c00605

[ref71] Jia W. L. Song C. He L. Y. Wang B. Gao F. Z. Zhang M.. (2023). Antibiotics in soil and water: occurrence, fate, and risk. Curr. Opin. Environ. Sci. Health 32:100437. doi: 10.1016/j.coesh.2022.100437

[ref72] Jorge N. L. Garrafa M. V. Romero J. M. Jorge M. J. Jorge L. C. Delfino M. R.. (2024). Adsorption of ciprofloxacin on clay minerals in Argentinian Santa Rosa-Corrientes soils. Molecules 29:1760. doi: 10.3390/molecules29081760, PMID: 38675580 PMC11051898

[ref73] Karigar C. S. Rao S. S. (2011). Role of microbial enzymes in the bioremediation of pollutants: a review. Enzyme Res. 2011:805187. doi: 10.4061/2011/805187, PMID: 21912739 PMC3168789

[ref74] Kaviani Rad A. Astaykina A. Streletskii R. Afsharyzad Y. Etesami H. Zarei M.. (2022). An overview of antibiotic resistance and abiotic stresses affecting antimicrobial resistance in agricultural soils. Int. J. Environ. Res. Public Health 19:4666. doi: 10.3390/ijerph19084666, PMID: 35457533 PMC9025980

[ref75] Keenum I. Williams R. K. Ray P. Garner E. D. Knowlton K. F. Pruden A. (2021). Combined effects of composting and antibiotic administration on cattle manure–borne antibiotic resistance genes. Microbiome 9:81. doi: 10.1186/s40168-021-01006-z, PMID: 33795006 PMC8017830

[ref76] Khan N. A. Ahmed S. Farooqi I. H. Ali I. Vambol V. Changani F.. (2020). Occurrence, sources and conventional treatment techniques for various antibiotics present in hospital wastewaters: a critical review. Trac-Trends Anal. Chem. 129:115921. doi: 10.1016/j.trac.2020.115921

[ref77] Khmaissa M. Zouari-Mechichi H. Sciara G. Record E. Mechichi T. (2024). Pollution from livestock farming antibiotics an emerging environmental and human health concern: a review. J. Hazard. Mater. Adv. 13:100410. doi: 10.1016/j.hazadv.2024.100410

[ref78] Kim J. J. Seong H. J. Johnson T. A. Cha C. J. Sul W. J. Chae J. C. (2023). Persistence of antibiotic resistance from animal agricultural effluents to surface water revealed by genome-centric metagenomics. J. Hazard. Mater. 457:131761. doi: 10.1016/j.jhazmat.2023.131761, PMID: 37290355

[ref79] Kodesova R. Svecova H. Klement A. Fer M. Nikodem A. Fedorova G.. (2024). Contamination of water, soil, and plants by micropollutants from reclaimed wastewater and sludge from a wastewater treatment plant. Sci. Total Environ. 907:167965. doi: 10.1016/j.scitotenv.2023.167965, PMID: 37866592

[ref80] Kovalakova P. Cizmas L. Mcdonald T. J. Marsalek B. Feng M. Sharma V. K. (2020). Occurrence and toxicity of antibiotics in the aquatic environment: a review. Chemosphere 251:126351. doi: 10.1016/j.chemosphere.2020.126351, PMID: 32443222

[ref81] Kraemer S. A. Ramachandran A. Perron G. G. (2019). Antibiotic pollution in the environment: from microbial ecology to public policy. Microorganisms 7:180. doi: 10.3390/microorganisms7060180, PMID: 31234491 PMC6616856

[ref82] Larsson D. G. J. Flach C. F. (2022). Antibiotic resistance in the environment. Nat. Rev. Microbiol. 20, 257–269. doi: 10.1038/s41579-021-00649-x, PMID: 34737424 PMC8567979

[ref83] Laxminarayan R. Chaudhury R. R. (2016). Antibiotic resistance in India: drivers and opportunities for action. PLoS Med. 13:e1001974. doi: 10.1371/journal.pmed.1001974, PMID: 26934098 PMC4775002

[ref84] Leiva A. M. Piña B. Vidal G. (2021). Antibiotic resistance dissemination in wastewater treatment plants: a challenge for the reuse of treated wastewater in agriculture. Rev. Environ. Sci. Biotechnol. 20, 1043–1072. doi: 10.1007/s11157-021-09588-8

[ref85] Leng Y. Bao J. Chang G. Zheng H. Li X. Du J.. (2016). Biotransformation of tetracycline by a novel bacterial strain *Stenotrophomonas maltophilia* DT1. J. Hazard. Mater. 318, 125–133. doi: 10.1016/j.jhazmat.2016.06.053, PMID: 27420384

[ref86] Li X. Bao N. Yan Z. Yuan X.-Z. Wang S.-G. Xia P.-F. (2023). Degradation of antibiotic resistance genes by VADER with CRISPR-Cas immunity. Appl. Environ. Microbiol. 89, e00053–e00023. doi: 10.1128/aem.00053-23, PMID: 36975789 PMC10132114

[ref87] Li S. Y. Hofstra N. Van De Schans M. G. M. Yang J. Li Y. A. Zhang Q.. (2023). Riverine antibiotics from animal production and wastewater. Environ. Sci. Technol. Lett. 10, 1059–1067. doi: 10.1007/s10661-023-11569-z

[ref88] Li H. Lin Y. Qin X. Song L. Fan F. Liu Y.. (2024). An updated review on how biochar may possess potential in soil ARGs control on aspects of source, fate and elimination. Biochar 6:24. doi: 10.1007/s42773-024-00319-0

[ref89] Li T. Xu J. Zhao X. Zhang Q. Zhu T. Fan D.. (2024). Impacts of irrigation with treated livestock wastewater on the accumulation characteristic of ARGs in the farmland soil: a case study in Hohhot, China. Environ. Geochem. Health 46:26. doi: 10.1007/s10653-023-01811-5, PMID: 38225519

[ref90] Li H. Z. Yang K. Liao H. Lassen S. B. Su J. Q. Zhang X.. (2022). Active antibiotic resistome in soils unraveled by single-cell isotope probing and targeted metagenomics. Proc. Natl. Acad. Sci. USA 119:e2201473119. doi: 10.1073/pnas.2201473119, PMID: 36161886 PMC9546533

[ref91] Li Q. Zheng Y. Guo L. Xiao Y. Li H. Yang P.. (2024). Microbial degradation of tetracycline antibiotics: mechanisms and environmental implications. J. Agric. Food Chem. 72, 13523–13536. doi: 10.1021/acs.jafc.4c02677, PMID: 38835142

[ref92] Liang Y. Pei M. Wang D. Cao S. Xiao X. Sun B. (2017). Improvement of soil ecosystem multifunctionality by dissipating manure-induced antibiotics and resistance genes. Environ. Sci. Technol. 51, 4988–4998. doi: 10.1021/acs.est.7b00693, PMID: 28394116

[ref93] Lima T. Domingues S. Da Silva G. J. (2020). Manure as a potential hotspot for antibiotic resistance dissemination by horizontal gene transfer events. Vet. Sci. 7:110. doi: 10.3390/vetsci7030110, PMID: 32823495 PMC7558842

[ref94] Lin Q. Yang Y. Zhang S. Sun F. Shen C. Su X. (2024). Enhanced biodegradation of polychlorinated biphenyls by co-cultivation of resuscitated strains with unique advantages. Environ. Res. 261:119699. doi: 10.1016/j.envres.2024.119699, PMID: 39074776

[ref95] Liu X. Chen J. Liu Y. Wan Z. Guo X. Lu S.. (2022a). Sulfamethoxazole degradation by *Pseudomonas silesiensis* F6a isolated from bioelectrochemical technology-integrated constructed wetlands. Ecotoxicol. Environ. Saf. 240:113698. doi: 10.1016/j.ecoenv.2022.113698, PMID: 35636241

[ref96] Liu X. Zhang J. Gbadegesin L. A. He Y. (2022b). Modelling approaches for linking the residual concentrations of antibiotics in soil with antibiotic properties and land-use types in the largest urban agglomerations in China: A review. Sci. Total Environ. 838:156141. doi: 10.1016/j.scitotenv.2022.156141, PMID: 35609696

[ref97] Looft T. Johnson T. A. Allen H. K. Bayles D. O. Alt D. P. Stedtfeld R. D.. (2012). In-feed antibiotic effects on the swine intestinal microbiome. Proc. Natl. Acad. Sci. USA 109, 1691–1696. doi: 10.1073/pnas.1120238109, PMID: 22307632 PMC3277147

[ref98] Lopez Gordillo A. P. Trueba-Santiso A. Lema J. M. Schäffer A. Smith K. E. C. (2024). Sulfamethoxazole is metabolized and mineralized at extremely low concentrations. Environ. Sci. Technol. 58, 9723–9730. doi: 10.1021/acs.est.4c02191, PMID: 38761139 PMC11155234

[ref99] Lu Y. Li J. Meng J. Zhang J. Zhuang H. Zheng G.. (2021). Long-term biogas slurry application increased antibiotics accumulation and antibiotic resistance genes (ARGs) spread in agricultural soils with different properties. Sci. Total Environ. 759:143473. doi: 10.1016/j.scitotenv.2020.143473, PMID: 33203566

[ref100] Luo T. Chen T. Cheng W. Lassabatere L. Boily J. F. Hanna K. (2024). Impact of water saturation on the fate and mobility of antibiotics in reactive porous Geomedia. Environ. Sci. Technol. 58, 15827–15835. doi: 10.1021/acs.est.4c06222, PMID: 39171685

[ref101] Luo H. Liu C. He D. Xu J. Sun J. Li J.. (2022). Environmental behaviors of microplastics in aquatic systems: A systematic review on degradation, adsorption, toxicity and biofilm under aging conditions. J. Hazard. Mater. 423:126915. doi: 10.1016/j.jhazmat.2021.126915, PMID: 34461541

[ref102] Lyu J. Yang L. Zhang L. Ye B. Wang L. (2020). Antibiotics in soil and water in China-a systematic review and source analysis. Environ. Pollut. 266:115147. doi: 10.1016/j.envpol.2020.115147, PMID: 32673932

[ref103] Mafiz A. He Y. Zhang W. Zhang Y. (2021). Soil bacteria in urban community gardens have the potential to disseminate antimicrobial resistance through horizontal gene transfer. Front. Microbiol. 12:771707. doi: 10.3389/fmicb.2021.771707, PMID: 34887843 PMC8650581

[ref104] Mangwani N. Kumari S. Das S. (2016). Bacterial biofilms and quorum sensing: fidelity in bioremediation technology. Biotechnol. Genet. Eng. Rev. 32, 43–73. doi: 10.1080/02648725.2016.1196554, PMID: 27320224

[ref105] Markley J. L. Wencewicz T. A. (2018). Tetracycline-inactivating enzymes. Front. Microbiol. 9:1058. doi: 10.3389/fmicb.2018.01058, PMID: 29899733 PMC5988894

[ref106] Matamoros V. Casas M. E. Pastor E. Tadic D. Canameras N. Carazo N.. (2022). Effects of tetracycline, sulfonamide, fluoroquinolone, and lincosamide load in pig slurry on lettuce: agricultural and human health implications. Environ. Res. 215:114237. doi: 10.1016/j.envres.2022.114237, PMID: 36084673

[ref107] Mayorga-Ramos A. Zúñiga-Miranda J. Carrera-Pacheco S. E. Barba-Ostria C. Guamán L. P. (2023). CRISPR-Cas-based antimicrobials: design, challenges, and bacterial mechanisms of resistance. ACS Infect. Dis. 9, 1283–1302. doi: 10.1021/acsinfecdis.2c00649, PMID: 37347230 PMC10353011

[ref108] Mceachran A. D. Blackwell B. R. Hanson J. D. Wooten K. J. Mayer G. D. Cox S. B.. (2015). Antibiotics, bacteria, and antibiotic resistance genes: aerial transport from cattle feed yards via particulate matter. Environ. Health Perspect. 123, 337–343. doi: 10.1289/ehp.1408555, PMID: 25633846 PMC4383574

[ref109] Mdegela R. H. Mwakapeje E. R. Rubegwa B. Gebeyehu D. T. Niyigena S. Msambichaka V.. (2021). Antimicrobial use, residues, resistance and governance in the food and agriculture sectors, Tanzania. Antibiotics (Basel) 10:454. doi: 10.3390/antibiotics10040454, PMID: 33923689 PMC8073917

[ref110] Mehanni M. M. Gadow S. I. Alshammari F. A. Modafer Y. Ghanem K. Z. El-Tahtawi N. F.. (2023). Antibiotic-resistant bacteria in hospital wastewater treatment plant effluent and the possible consequences of its reuse in agricultural irrigation. Front. Microbiol. 14:1141383. doi: 10.3389/fmicb.2023.1141383, PMID: 37143530 PMC10153669

[ref111] Mohy U. D. N. Farhan M. Wahid A. Ciric L. Sharif F. (2023). Human health risk estimation of antibiotics transferred from wastewater and soil to crops. Environ. Sci. Pollut. Res. Int. 30, 20601–20614. doi: 10.1007/s11356-022-23412-y, PMID: 36255570

[ref112] Mokrani S. Houali K. Yadav K. K. Arabi A. I. A. Eltayeb L. B. Awjanalreshidi M.. (2024). Bioremediation techniques for soil organic pollution: mechanisms, microorganisms, and technologies - a comprehensive review. Ecol. Eng. 207:107338. doi: 10.1016/j.ecoleng.2024.107338

[ref113] Molale-Tom L. G. Olanrewaju O. S. Kritzinger R. K. Fri J. Bezuidenhout C. C. (2024). Heterotrophic bacteria in drinking water: evaluating antibiotic resistance and the presence of virulence genes. Microbiol. Spectr. 12:e0335923. doi: 10.1128/spectrum.03359-23, PMID: 38205959 PMC10845987

[ref114] Muhammad J. Khan S. Su J. Q. Hesham A. Ditta A. Nawab J.. (2020). Antibiotics in poultry manure and their associated health issues: a systematic review. J. Soils Sediments 20, 486–497. doi: 10.1007/s11368-019-02360-0

[ref115] Mulla S. I. Hu A. Sun Q. Li J. Suanon F. Ashfaq M.. (2018). Biodegradation of sulfamethoxazole in bacteria from three different origins. J. Environ. Manag. 206, 93–102. doi: 10.1016/j.jenvman.2017.10.029, PMID: 29059576

[ref116] Muurinen J. Stedtfeld R. Karkman A. Parnanen K. Tiedje J. Virta M. (2017). Influence of manure application on the environmental Resistome under Finnish agricultural practice with restricted antibiotic use. Environ. Sci. Technol. 51, 5989–5999. doi: 10.1021/acs.est.7b00551, PMID: 28453251

[ref117] Negreanu Y. Pasternak Z. Jurkevitch E. Cytryn E. (2012). Impact of treated wastewater irrigation on antibiotic resistance in agricultural soils. Environ. Sci. Technol. 46, 4800–4808. doi: 10.1021/es204665b, PMID: 22494147

[ref118] Neil K. Allard N. Roy P. Grenier F. Menendez A. Burrus V.. (2021). High-efficiency delivery of CRISPR-Cas9 by engineered probiotics enables precise microbiome editing. Mol. Syst. Biol. 17:e10335. doi: 10.15252/msb.202110335, PMID: 34665940 PMC8527022

[ref119] Nkoh J. N. Shang C. Okeke E. S. Ejeromedoghene O. Oderinde O. Etafo N. O.. (2024). Antibiotics soil-solution chemistry: A review of environmental behavior and uptake and transformation by plants. J. Environ. Manag. 354:120312. doi: 10.1016/j.jenvman.2024.120312, PMID: 38340667

[ref120] Okoli A. S. Blix T. Myhr A. I. Xu W. Xu X. (2022). Sustainable use of CRISPR/Cas in fish aquaculture: the biosafety perspective. Transgenic Res. 31, 1–21. doi: 10.1007/s11248-021-00274-7, PMID: 34304349 PMC8821480

[ref121] Olanrewaju O. S. Glick B. R. Babalola O. O. (2017). Mechanisms of action of plant growth promoting bacteria. World J. Microbiol. Biotechnol. 33:197. doi: 10.1007/s11274-017-2364-9, PMID: 28986676 PMC5686270

[ref122] Olanrewaju O. S. Glick B. R. Babalola O. O. (2024a). Metabolomics-guided utilization of beneficial microbes for climate-resilient crops. Curr. Opin. Chem. Biol. 79:102427. doi: 10.1016/j.cbpa.2024.102427, PMID: 38290195

[ref123] Olanrewaju O. S. Molale-Tom L. G. Bezuidenhout C. C. (2024b). Genomic diversity, antibiotic resistance, and virulence in south African *Enterococcus faecalis* and *Enterococcus lactis* isolates. World J. Microbiol. Biotechnol. 40:289. doi: 10.1007/s11274-024-04098-5, PMID: 39102038 PMC11300488

[ref124] Olanrewaju O. S. Molale-Tom L. G. Kritzinger R. K. Bezuidenhout C. C. (2024c). Genome mining of *Escherichia coli* WG5D from drinking water source: unraveling antibiotic resistance genes, virulence factors, and pathogenicity. BMC Genomics 25:263. doi: 10.1186/s12864-024-10110-x, PMID: 38459466 PMC10924361

[ref125] Osbiston K. Oxbrough A. Fernandez-Martinez L. T. (2021). Antibiotic resistance levels in soils from urban and rural land uses in Great Britain. Access Microbiol. 3:acmi000181. doi: 10.1099/acmi.0.000181, PMID: 33997612 PMC8115975

[ref126] Pan M. Zhang H. Luo L.-W. Yau P.-C. (2023). Exploring the potential of co-application of sewage sludge, Chinese medicinal herbal residues and biochar in minimizing human exposure to antibiotics contamination in edible crops. Sustainability 15:2980. doi: 10.3390/su15042980

[ref127] Paris L. Devers-Lamrani M. Joly M. Viala D. De Antonio M. Pereira B.. (2023). Effect of subtherapeutic and therapeutic sulfamethazine concentrations on transcribed genes and translated proteins involved in *Microbacterium* sp. C448 resistance and degradation. FEMS Microbiol. Ecol. 99:fiad064. doi: 10.1093/femsec/fiad064 37309049

[ref128] Pastor-Lopez E. J. Casas M. E. Hellman D. Müller J. A. Matamoros V. (2024). Nature-based solutions for antibiotics and antimicrobial resistance removal in tertiary wastewater treatment: microbiological composition and risk assessment. Water Res. 261:122038. doi: 10.1016/j.watres.2024.122038, PMID: 38996727

[ref129] Pauletto M. De Liguoro M. (2024). A review on fluoroquinolones’ toxicity to freshwater organisms and a risk assessment. J. Xenobiot. 14, 717–752. doi: 10.3390/jox14020042, PMID: 38921651 PMC11205205

[ref130] Pepper I. L. Brooks J. P. Gerba C. P. (2018). Antibiotic resistant Bacteria in municipal wastes: is there reason for concern? Environ. Sci. Technol. 52, 3949–3959. doi: 10.1021/acs.est.7b04360, PMID: 29505255

[ref131] Phan D. Bhattacharjee A. S. Hanan D. Park S. Herrera D. Ashworth D.. (2024). Dissemination of antimicrobial resistance in agricultural ecosystems following irrigation with treated municipal wastewater. Sci. Total Environ. 934:173288. doi: 10.1016/j.scitotenv.2024.173288, PMID: 38768725

[ref132] Piazza A. Ciancio Casalini L. Pacini V. A. Sanguinetti G. Ottado J. Gottig N. (2019). Environmental Bacteria involved in manganese(II) oxidation and removal from groundwater. Front. Microbiol. 10:119. doi: 10.3389/fmicb.2019.00119, PMID: 30853942 PMC6396730

[ref133] Qian X. Wang Z. Zhang H. Gu H. Shen G. (2022). Occurrence of veterinary antibiotics in animal manure, compost, and agricultural soil, originating from different feedlots in suburbs of Shanghai, East China. Environ. Monit. Assess. 194:379. doi: 10.1007/s10661-022-10010-1, PMID: 35441264

[ref134] Qin X. Zhai L. Khoshnevisan B. Pan J. Liu H. (2022). Restriction of biosolids returning to land: fate of antibiotic resistance genes in soils after long-term biosolids application. Environ. Pollut. 301:119029. doi: 10.1016/j.envpol.2022.119029, PMID: 35217140

[ref135] Qiu J. Chen Y. Feng Y. Li X. Xu J. Jiang J. (2023). Adaptation of rhizosphere microbial communities to continuous exposure to multiple residual antibiotics in vegetable farms. Int. J. Environ. Res. Public Health 20:3137. doi: 10.3390/ijerph20043137, PMID: 36833828 PMC9958589

[ref136] Rahman M. Alam M. U. Luies S. K. Kamal A. Ferdous S. Lin A.. (2021). Contamination of fresh produce with antibiotic-resistant bacteria and associated risks to human health: a scoping review. Int. J. Environ. Res. Public Health 19:360. doi: 10.3390/ijerph19010360, PMID: 35010620 PMC8744955

[ref137] Ray P. Chen C. Knowlton K. F. Pruden A. Xia K. (2017). Fate and effect of antibiotics in beef and dairy manure during static and turned composting. J. Environ. Qual. 46, 45–54. doi: 10.2134/jeq2016.07.0269, PMID: 28177414

[ref138] Ren J. Qi X. Zhang J. Niu D. Shen Y. Yu C.. (2023). Biodegradation efficiency and mechanism of erythromycin degradation by *Paracoccus versutus* W7. J. Environ. Manag. 332:117372. doi: 10.1016/j.jenvman.2023.117372, PMID: 36731410

[ref139] Ricken B. Kolvenbach B. A. Bergesch C. Benndorf D. Kroll K. Strnad H.. (2017). FMNH2-dependent monooxygenases initiate catabolism of sulfonamides in *Microbacterium* sp. strain BR1 subsisting on sulfonamide antibiotics. Sci. Rep. 7:15783. doi: 10.1038/s41598-017-16132-8, PMID: 29150672 PMC5693940

[ref140] Rodrigues D. Da Cunha C. Pereira A. R. Espirito Santo D. R. D. Silva S. Q. Starling M.. (2025). Biodegradation of trimethoprim and sulfamethoxazole in secondary effluent by microalgae-bacteria consortium. Int. J. Hyg. Environ. Health 264:114517. doi: 10.1016/j.ijheh.2024.114517, PMID: 39724811

[ref141] Rodríguez-López L. Santás-Miguel V. Cela-Dablanca R. Núñez-Delgado A. Álvarez-Rodríguez E. Rodríguez-Seijo A.. (2024). Sorption of antibiotics in agricultural soils as a function of pH. Span. J. Soil Sci. 14:12402. doi: 10.3389/sjss.2024.12402

[ref142] Rossi F. Rizzotti L. Felis G. E. Torriani S. (2014). Horizontal gene transfer among microorganisms in food: current knowledge and future perspectives. Food Microbiol. 42, 232–243. doi: 10.1016/j.fm.2014.04.004, PMID: 24929742

[ref143] Samreen Ahmad I. Malak H. A. Abulreesh H. H. (2021). Environmental antimicrobial resistance and its drivers: a potential threat to public health. J. Glob. Antimicrob. Resist. 27, 101–111. doi: 10.1016/j.jgar.2021.08.001, PMID: 34454098

[ref144] Sanderson C. E. Fox J. T. Dougherty E. R. Cameron A. D. S. Alexander K. A. (2018). The changing face of water: A dynamic reflection of antibiotic resistance across landscapes. Front. Microbiol. 9:1894. doi: 10.3389/fmicb.2018.01894, PMID: 30237787 PMC6135886

[ref145] Santás-Miguel V. Díaz-Raviña M. Martín A. García-Campos E. Barreiro A. Núñez-Delgado A.. (2021). Soil enzymatic activities and microbial community structure in soils polluted with tetracycline antibiotics. Agronomy 11:906. doi: 10.3390/agronomy11050906

[ref146] Sara M. N. (2003). Site assessment and remediation handbook. Boca Raton, Florida: CRC Press.

[ref147] Scaccia N. Vaz-Moreira I. Manaia C. M. (2021). The risk of transmitting antibiotic resistance through endophytic bacteria. Trends Plant Sci. 26, 1213–1226. doi: 10.1016/j.tplants.2021.09.001, PMID: 34593300

[ref148] Shao S. Hu Y. Cheng J. Chen Y. (2019). Biodegradation mechanism of tetracycline (TEC) by strain *Klebsiella* sp. SQY5 as revealed through products analysis and genomics. Ecotoxicol. Environ. Saf. 185:109676. doi: 10.1016/j.ecoenv.2019.109676, PMID: 31539769

[ref149] Shao S. Hu Y. Cheng C. Cheng J. Chen Y. (2018). Simultaneous degradation of tetracycline and denitrification by a novel bacterium, *Klebsiella* sp. SQY5. Chemosphere 209, 35–43. doi: 10.1016/j.chemosphere.2018.06.093, PMID: 29913397

[ref150] Sharma P. Poustie A. Verburg P. Pagilla K. Yang Y. Hanigan D. (2020). Trace organic contaminants in field-scale cultivated alfalfa, soil, and pore water after 10 years of irrigation with reclaimed wastewater. Sci. Total Environ. 744:140698. doi: 10.1016/j.scitotenv.2020.140698, PMID: 32693273

[ref151] Shawver S. Ishii S. Strickland M. S. Badgley B. (2024). Soil type and moisture content alter soil microbial responses to manure from cattle administered antibiotics. Environ. Sci. Pollut. Res. 31, 27259–27272. doi: 10.1007/s11356-024-32903-z, PMID: 38507165 PMC11052774

[ref152] Shawver S. Wepking C. Ishii S. Strickland M. S. Badgley B. D. (2021). Application of manure from cattle administered antibiotics has sustained multi-year impacts on soil resistome and microbial community structure. Soil Biol. Biochem. 157:108252. doi: 10.1016/j.soilbio.2021.108252

[ref153] Shi B. S. Cheng X. J. Chen H. Z. Xie J. Zhou Z. H. Jiang S. Q.. (2022). Occurrence, source tracking and removal of antibiotics in recirculating aquaculture systems (RAS) in southern China. J. Environ. Manag. 324:116311. doi: 10.1016/j.jenvman.2022.116311, PMID: 36162319

[ref154] Shu Y. Li D. Xie T. Zhao K. Zhou L. Li F. (2025). Antibiotics-heavy metals combined pollution in agricultural soils: sources, fate, risks, and countermeasures. Green Energy Environ. 10, 869–897. doi: 10.1016/j.gee.2024.07.007

[ref155] Shun-Mei E. Zeng J.-M. Yuan H. Lu Y. Cai R.-X. Chen C. (2018). Sub-inhibitory concentrations of fluoroquinolones increase conjugation frequency. Microb. Pathog. 114, 57–62. doi: 10.1016/j.micpath.2017.11.036, PMID: 29174700

[ref156] Singh J. S. Abhilash P. C. Singh H. B. Singh R. P. Singh D. P. (2011). Genetically engineered bacteria: an emerging tool for environmental remediation and future research perspectives. Gene 480, 1–9. doi: 10.1016/j.gene.2011.03.001, PMID: 21402131

[ref157] Snow D. D. Cassada D. A. Biswas S. Malakar A. D'alessio M. Marshall A. H. L.. (2020). Detection, occurrence, and fate of emerging contaminants in agricultural environments (2020). Water Environ. Res. 92, 1741–1750. doi: 10.1002/wer.1429, PMID: 32762100

[ref158] Sonawane J. M. Rai A. K. Sharma M. Tripathi M. Prasad R. (2022). Microbial biofilms: recent advances and progress in environmental bioremediation. Sci. Total Environ. 824:153843. doi: 10.1016/j.scitotenv.2022.153843, PMID: 35176385

[ref159] Sorinolu A. J. Tyagi N. Kumar A. Munir M. (2021). Antibiotic resistance development and human health risks during wastewater reuse and biosolids application in agriculture. Chemosphere 265:129032. doi: 10.1016/j.chemosphere.2020.129032, PMID: 33293048

[ref160] Storteboom H. Arabi M. Davis J. G. Crimi B. Pruden A. (2010). Tracking antibiotic resistance genes in the South Platte River basin using molecular signatures of urban, agricultural, and pristine sources. Environ. Sci. Technol. 44, 7397–7404. doi: 10.1021/es101657s, PMID: 20809616

[ref161] Sudha S. Parthasarathi N. Prabha D. Velmurugan P. Sivakumar S. Anitha V.. (2022). Oxytetracycline degrading potential of *Lysinibacillus* sp. strain 3+I isolated from poultry manure. Evid. Based Complement. Alternat. Med. 2022:2750009. doi: 10.1155/2022/2750009, PMID: 35368761 PMC8970894

[ref162] Sun Y. Snow D. Walia H. Li X. (2021). Transmission routes of the microbiome and Resistome from manure to soil and lettuce. Environ. Sci. Technol. 55, 11102–11112. doi: 10.1021/acs.est.1c02985, PMID: 34323079

[ref163] Sun Y. Staley Zachery R. Woodbury B. Riethoven J.-J. Li X. (2024). Composting reduces the risks of resistome in beef cattle manure at the transcriptional level. Appl. Environ. Microbiol. 90, e01752–e01723. doi: 10.1128/aem.01752-23, PMID: 38445903 PMC11022583

[ref164] Sutherland I. (2001). Biofilm exopolysaccharides: a strong and sticky framework. Microbiology (Reading) 147, 3–9. doi: 10.1099/00221287-147-1-3, PMID: 11160795

[ref165] Syed A. Zeyad M. T. Shahid M. Elgorban A. M. Alkhulaifi M. M. Ansari I. A. (2021). Heavy metals induced modulations in growth, physiology, cellular viability, and biofilm formation of an identified bacterial isolate. ACS Omega 6, 25076–25088. doi: 10.1021/acsomega.1c04396, PMID: 34604686 PMC8482775

[ref166] Tadic D. Bleda Hernandez M. J. Cerqueira F. Matamoros V. Pina B. Bayona J. M. (2021). Occurrence and human health risk assessment of antibiotics and their metabolites in vegetables grown in field-scale agricultural systems. J. Hazard. Mater. 401:123424. doi: 10.1016/j.jhazmat.2020.123424, PMID: 33113716

[ref167] Tan Z. Yang X. Liu Y. Chen L. Xu H. Li Y.. (2023). The capability of chloramphenicol biotransformation of *Klebsiella* sp. YB1 under cadmium stress and its genome analysis. Chemosphere 313:137375. doi: 10.1016/j.chemosphere.2022.137375, PMID: 36435315

[ref168] Tao S. Chen H. Li N. Liang W. (2022). The application of the CRISPR-Cas system in antibiotic resistance. Infect. Drug Resist. 15, 4155–4168. doi: 10.2147/IDR.S370869, PMID: 35942309 PMC9356603

[ref169] Taylor P. Reeder R. (2020). Antibiotic use on crops in low and middle-income countries based on recommendations made by agricultural advisors. CABI Agric. Biosci. 1:1. doi: 10.1186/s43170-020-00001-y, PMID: 40926190

[ref170] Thiang E. L. Lee C. W. Takada H. Seki K. Takei A. Suzuki S.. (2021). Antibiotic residues from aquaculture farms and their ecological risks in Southeast Asia: a case study from Malaysia. Ecosyst. Health Sustain. 7:1926337. doi: 10.1080/20964129.2021.1926337

[ref171] Tian L. Sun H. Dong X. Wang J. Huang Y. Sun S. (2021). Effects of swine wastewater irrigation on soil properties and accumulation of heavy metals and antibiotics. J. Soils Sediments 22, 889–904. doi: 10.1007/s11368-021-03106-7

[ref172] Tiedje J. M. Fu Y. H. Mei Z. Schaeffer A. Dou Q. Y. Amelung W.. (2023). Antibiotic resistance genes in food production systems support one health opinions. Curr. Opin. Environ. Sci. Health 34:100492. doi: 10.1016/j.coesh.2023.100492

[ref173] Topp E. Larsson D. G. J. Miller D. N. Van Den Eede C. Virta M. P. J. (2018). Antimicrobial resistance and the environment: assessment of advances, gaps and recommendations for agriculture, aquaculture and pharmaceutical manufacturing. FEMS Microbiol. Ecol. 94:185. doi: 10.1093/femsec/fix185, PMID: 29309580

[ref174] Tyagi M. Da Fonseca M. M. De Carvalho C. C. (2011). Bioaugmentation and biostimulation strategies to improve the effectiveness of bioremediation processes. Biodegradation 22, 231–241. doi: 10.1007/s10532-010-9394-4, PMID: 20680666

[ref175] Udikovic-Kolic N. Wichmann F. Broderick N. A. Handelsman J. (2014). Bloom of resident antibiotic-resistant bacteria in soil following manure fertilization. Proc. Natl. Acad. Sci. USA 111, 15202–15207. doi: 10.1073/pnas.1409836111, PMID: 25288759 PMC4210343

[ref176] Ugulu I. Khan Z. I. Bibi S. Ahmad K. Munir M. Memona H. (2024). Evaluation of the effects of wastewater irrigation on heavy metal accumulation in vegetables and human health in the cauliflower example: heavy metal accumulation in cauliflower. Bull. Environ. Contam. Toxicol. 112:44. doi: 10.1007/s00128-024-03858-1, PMID: 38416161

[ref177] Urra J. Alkorta I. Lanzén A. Mijangos I. Garbisu C. (2019). The application of fresh and composted horse and chicken manure affects soil quality, microbial composition and antibiotic resistance. Appl. Soil Ecol. 135, 73–84. doi: 10.1016/j.apsoil.2018.11.005

[ref178] Valentine D. L. Kessler J. D. Redmond M. C. Mendes S. D. Heintz M. B. Farwell C.. (2010). Propane respiration jump-starts microbial response to a deep oil spill. Science 330, 208–211. doi: 10.1126/science.1196830, PMID: 20847236

[ref179] Verhaegen M. Bergot T. Liebana E. Stancanelli G. Streissl F. Mingeot-Leclercq M. P.. (2023). On the use of antibiotics to control plant pathogenic bacteria: a genetic and genomic perspective. Front. Microbiol. 14:1221478. doi: 10.3389/fmicb.2023.1221478, PMID: 37440885 PMC10333595

[ref180] Vestel J. Caldwell D. J. Tell J. Constantine L. Häner A. Hellstern J.. (2022). Default predicted no-effect target concentrations for antibiotics in the absence of data for the protection against antibiotic resistance and environmental toxicity. Integr. Environ. Assess. Manag. 18, 863–867. doi: 10.1002/ieam.4560, PMID: 34826209 PMC9302680

[ref181] Wang G. Gao X. Cai Y. Li G. Ma R. Yuan J. (2024). Dynamics of antibiotic resistance genes during manure composting: reduction in herbivores manure and accumulation in carnivores. Environ. Int. 190:108900. doi: 10.1016/j.envint.2024.108900, PMID: 39053194

[ref182] Wang S. Li W. Xi B. Cao L. Huang C. (2024). Mechanisms and influencing factors of horizontal gene transfer in composting system: A review. Sci. Total Environ. 955:177017. doi: 10.1016/j.scitotenv.2024.177017, PMID: 39427888

[ref183] Wang Q. Wang H. Lv M. Wang X. Chen L. (2023). Sulfamethoxazole degradation by Aeromonas caviae and co-metabolism by the mixed bacteria. Chemosphere 317:137882. doi: 10.1016/j.chemosphere.2023.137882, PMID: 36657578

[ref184] Wang E. Yu B. Zhang J. Gu S. Yang Y. Deng Y.. (2024). Low carbon loss from long-term manure-applied soil during abrupt warming is realized through soil and microbiome interplay. Environ. Sci. Technol. 58, 9658–9668. doi: 10.1021/acs.est.3c08319 38768036

[ref185] Wei Z. Gu Y. Friman V. P. Kowalchuk G. A. Xu Y. Shen Q.. (2019). Initial soil microbiome composition and functioning predetermine future plant health. Sci. Adv. 5:eaaw0759. doi: 10.1126/sciadv.aaw0759, PMID: 31579818 PMC6760924

[ref186] Wepking C. Avera B. Badgley B. Barrett J. E. Franklin J. Knowlton K. F.. (2017). Exposure to dairy manure leads to greater antibiotic resistance and increased mass-specific respiration in soil microbial communities. Proc. R. Soc. B Biol. Sci. 284:20162233. doi: 10.1098/rspb.2016.2233, PMID: 28356447 PMC5378074

[ref187] Wepking C. Badgley B. Barrett J. E. Knowlton K. F. Lucas J. M. Minick K. J.. (2019). Prolonged exposure to manure from livestock-administered antibiotics decreases ecosystem carbon-use efficiency and alters nitrogen cycling. Ecol. Lett. 22, 2067–2076. doi: 10.1111/ele.13390, PMID: 31595680

[ref188] Williams-Nguyen J. Sallach J. B. Bartelt-Hunt S. Boxall A. B. Durso L. M. Mclain J. E.. (2016). Antibiotics and antibiotic resistance in agroecosystems: state of the science. J. Environ. Qual. 45, 394–406. doi: 10.2134/jeq2015.07.0336, PMID: 27065386

[ref189] Wind L. Krometis L.-A. Hession W. C. Chen C. Du P. Jacobs K.. (2018). Fate of Pirlimycin and antibiotic-resistant fecal coliforms in field plots amended with dairy manure or compost during vegetable cultivation. J. Environ. Qual. 47, 436–444. doi: 10.2134/jeq2017.12.0491, PMID: 29864178

[ref190] Wu J. Wang J. Li Z. Guo S. Li K. Xu P.. (2022). Antibiotics and antibiotic resistance genes in agricultural soils: a systematic analysis. Crit. Rev. Environ. Sci. Technol. 53, 847–864. doi: 10.1080/10643389.2022.2094693

[ref191] Xiao W. Zhao X. Teng Y. Wu J. Zhang T. (2023). Review on biogeochemical characteristics of typical antibiotics in groundwater in China. Sustainability 15:6985. doi: 10.3390/su15086985

[ref192] Xie W. Y. Shen Q. Zhao F. J. (2018). Antibiotics and antibiotic resistance from animal manures to soil: a review. Eur. J. Soil Sci. 69, 181–195. doi: 10.1111/ejss.12494

[ref193] Xu H. Chen Z. Huang R. Cui Y. Li Q. Zhao Y.. (2021). Antibiotic resistance gene-carrying plasmid spreads into the plant endophytic Bacteria using soil Bacteria as carriers. Environ. Sci. Technol. 55, 10462–10470. doi: 10.1021/acs.est.1c01615, PMID: 34114802

[ref194] Xu X. Y. Ji F. Zhuang J. L. Cui J. H. Huang T. Y. Zhang M. L.. (2024). Enhanced removal of PHE-Cd2+co-contamination by the mixed bacterial cultures of *Pseudomonas putida* and *Arthrobacter* sp.: performance and mechanism. Biochem. Eng. J. 210:109433. doi: 10.1016/j.bej.2024.109433

[ref195] Xu J. Wang B. Zhang W. H. Zhang F. J. Deng Y. D. Wang Y.. (2021). Biodegradation of p-nitrophenol by engineered strain. AMB Express 11:124. doi: 10.1186/s13568-021-01284-8, PMID: 34463855 PMC8408293

[ref196] Yang Q. Zhang H. Guo Y. Tian T. (2016). Influence of chicken manure fertilization on antibiotic-resistant Bacteria in soil and the endophytic Bacteria of Pakchoi. Int. J. Environ. Res. Public Health 13:662. doi: 10.3390/ijerph13070662, PMID: 27376311 PMC4962203

[ref197] Yang K. F. Zhao F. K. Yang L. Huang Y. Shen L. J. Liu H. L.. (2024). Occurrence and dissipation of antibiotics in manure-amended vegetable greenhouse soils under sprinkling irrigation. Environ. Res. Commun. 6:125024. doi: 10.1088/2515-7620/ad9e8d

[ref198] Yi N. Z. Wu S. Y. Su H. C. Hu X. J. Xu W. J. Xu Y.. (2025). Temporal and spatial changes, bioaccumulation, critical influencers, and environmental fate of antibiotics in small-scale greenhouse shrimp farming system. J. Environ. Chem. Eng. 13:115574. doi: 10.1016/j.jece.2025.115574

[ref199] Yin L. Wang X. Li Y. Liu Z. Mei Q. Chen Z. (2023). Uptake of the plant agriculture-used antibiotics oxytetracycline and streptomycin by cherry radish horizontal line effect on plant microbiome and the potential health risk. J. Agric. Food Chem. 71, 4561–4570. doi: 10.1021/acs.jafc.3c01052, PMID: 36945880

[ref200] Yuan S. Wang Z. Yuan S. (2024). Insights into the pH-dependent interactions of sulfadiazine antibiotic with soil particle models. Sci. Total Environ. 917:170537. doi: 10.1016/j.scitotenv.2024.170537, PMID: 38301792

[ref201] Yuan X. X. Zhang Y. Fan L. X. Wang W. B. Wu Y. J. (2022). Temporal dynamics of antibiotic resistance genes in vegetable greenhouse soils following different manure applications. J. Soil Sci. Plant Nutr. 22, 5144–5158. doi: 10.1007/s42729-022-00990-x

[ref202] Zalewska M. Blazejewska A. Czapko A. Popowska M. (2021). Antibiotics and antibiotic resistance genes in animal manure - consequences of its application in agriculture. Front. Microbiol. 12:610656. doi: 10.3389/fmicb.2021.610656, PMID: 33854486 PMC8039466

[ref203] Zeng Z. Liu Y. Zhong H. Xiao R. Zeng G. Liu Z.. (2018). Mechanisms for rhamnolipids-mediated biodegradation of hydrophobic organic compounds. Sci. Total Environ. 634, 1–11. doi: 10.1016/j.scitotenv.2018.03.349, PMID: 29625372

[ref204] Zha Y. Li Q. H. Liu H. Ge Y. Wei Y. H. Wang H. H.. (2023). Occurrence and ecological risk assessment of antibiotics in manure and the surrounding soil from typical chicken farms in Hangzhou, China. Front. Environ. Sci. 11:1241405. doi: 10.3389/fenvs.2023.1241405

[ref205] Zhang M. Fan D. Su C. Pan L. He Q. Li Z.. (2023). Biotransformation of sulfamethoxazole by a novel strain, *Nitratireductor* sp. GZWM139: characterized performance, metabolic mechanism and application potential. J. Hazard. Mater. 441:129861. doi: 10.1016/j.jhazmat.2022.129861, PMID: 36063713

[ref206] Zhang Y. Hao X. Thomas B. W. Mcallister T. A. Workentine M. Jin L.. (2023). Soil antibiotic resistance genes accumulate at different rates over four decades of manure application. J. Hazard. Mater. 443:130136. doi: 10.1016/j.jhazmat.2022.130136, PMID: 36444046

[ref207] Zhang M. He L. Y. Liu Y. S. Zhao J. L. Liu W. R. Zhang J. N.. (2019). Fate of veterinary antibiotics during animal manure composting. Sci. Total Environ. 650, 1363–1370. doi: 10.1016/j.scitotenv.2018.09.147, PMID: 30308823

[ref208] Zhang Y. J. Hu H. W. Chen Q. L. Singh B. K. Yan H. Chen D.. (2019). Transfer of antibiotic resistance from manure-amended soils to vegetable microbiomes. Environ. Int. 130:104912. doi: 10.1016/j.envint.2019.104912, PMID: 31220751

[ref209] Zhang T. Xu S.-Y. Lin H. Yang J. Zhao Z.-Q. Barceló D.. (2022). Efficient degradation of tylosin by *Klebsiella oxytoca* TYL-T1. Sci. Total Environ. 847:157305. doi: 10.1016/j.scitotenv.2022.157305, PMID: 35839875

[ref210] Zhang Y. Zhao Y.-G. Maqbool F. Hu Y. (2022). Removal of antibiotics pollutants in wastewater by UV-based advanced oxidation processes: influence of water matrix components, processes optimization and application: a review. J Water Process Eng 45:102496. doi: 10.1016/j.jwpe.2021.102496

[ref211] Zhang X. Zhu R. Li W. Ma J. Lin H. (2021). Genomic insights into the antibiotic resistance pattern of the tetracycline-degrading bacterium, *Arthrobacter nicotianae* OTC-16. Sci. Rep. 11:15638. doi: 10.1038/s41598-021-94840-y, PMID: 34341372 PMC8329189

[ref212] Zhao B. Van Bodegom P. M. Trimbos K. B. (2023). Antibiotic resistance genes in interconnected surface waters as affected by agricultural activities. Biomolecules 13:231. doi: 10.3390/biom13020231, PMID: 36830600 PMC9953135

[ref213] Zheng J. Zhang J. Gao L. Kong F. Shen G. Wang R.. (2020). The effects of tetracycline residues on the microbial community structure of tobacco soil in pot experiment. Sci. Rep. 10:8804. doi: 10.1038/s41598-020-65203-w, PMID: 32472015 PMC7260358

[ref214] Zhou S. Jia Y. Fang H. Jin C. Mo Y. Xiao Z.. (2024). A new understanding on the prerequisite of antibiotic biodegradation in wastewater treatment: adhesive behavior between antibiotic-degrading bacteria and ciprofloxacin. Water Res. 252:121226. doi: 10.1016/j.watres.2024.121226, PMID: 38309071

[ref215] Zhu L. Chu Y. Y. Liu F. Ai K. Y. Xu W. W. Cheng Y. Y.. (2025). Bacteria-supported manganese oxide for enhanced fluoroquinolone antibiotics removal in peroxymonosulfate oxidation system. Environ. Res. 270:121002. doi: 10.1016/j.envres.2025.121002, PMID: 39894150

[ref216] Zhu Y. G. Johnson T. A. Su J. Q. Qiao M. Guo G. X. Stedtfeld R. D.. (2013). Diverse and abundant antibiotic resistance genes in Chinese swine farms. Proc. Natl. Acad. Sci. USA 110, 3435–3440. doi: 10.1073/pnas.1222743110, PMID: 23401528 PMC3587239

[ref217] Zhu D. Xiang Q. Yang X. R. Ke X. O'connor P. Zhu Y. G. (2019). Trophic transfer of antibiotic resistance genes in a soil detritus food chain. Environ. Sci. Technol. 53, 7770–7781. doi: 10.1021/acs.est.9b00214, PMID: 31244079

